# What nudges you to take a vaccine? Understanding behavioural drivers of COVID-19 vaccinations using large-scale experiments in the G-7 countries

**DOI:** 10.1080/21642850.2025.2490550

**Published:** 2025-04-16

**Authors:** Manu M. Savani, Sanchayan Banerjee, Andrew Hunter, Peter John, Richard Koenig, Blake Lee-Whiting, Peter Loewen, John McAndrews, Brendan Nyhan

**Affiliations:** a Brunel University of London, London, UK; b King’s College London, London, UK; c Royal Holloway University London, Egham, UK; d University of Toronto, Toronto, Canada; e Cornell University, Ithaca, NY, USA; f McMaster University, Hamilton, Canada; g Dartmouth College, Hanover, NH, USA

**Keywords:** COVID-19, vaccination, policy support, nudge, nudge plus, conjoint experiments, survey experiments

## Abstract

**Introduction:**

We present a unique multi-country, two-wave dataset of 42,417 survey responses drawn from nationally representative samples of citizens from the G-7 countries: Canada, France, Germany, Italy, Japan, UK, and USA. This data note outlines the motivation and methodology of the survey instrument and describes the measures contained in the dataset. We highlight areas for future research.

**Methods:**

We fielded an online survey over two waves (January 27 to February 26 [*n* = 24,303] and wave 2 from March 6 to May 12 [*n* = 18,114]) measuring a range of demographic, social, political, and psychological variables. Samples were nationally representative by age, education, gender, and subnational region. Each wave included of three experiments (one conjoint and two between-subjects) to facilitate randomised evaluation of behavioural health policies promoting the uptake of COVID-19 booster vaccinations.

**Results:**

The dataset has produced two peer-reviewed publications at the time of writing ([Banerjee, S., John, P., Nyhan, B., Hunter, A., Koenig, R., Lee-Whiting, B., Loewen, P. J., McAndrews, J., & Savani, M. M. (2024). Thinking about default enrollment lowers vaccination intentions and public support in G7 countries. *PNAS Nexus*, *3*(4), pgae093]; [Koenig, R., Savani, M. M., Lee-Whiting, B., McAndrews, J., Banerjee, S., Hunter, A., John, P., Loewen, P. J., & Nyhan, B. (2024). Public support for more stringent vaccine policies increases with vaccine effectiveness. *Scientific Reports*, *14*(1), 1748]). A summary report is posted online (https://www.thebritishacademy.ac.uk/publications/overcoming-barriers-to-vaccination-by-empowering-citizens-to-make-deliberate-choices/). Additional research outputs are currently under preparation.

**Discussion:**

Our dataset combines observational and experimental data on behavioural health policies, offering numerous insights. First, the dataset's extensive size and geographical diversity enables comparative analysis of public health issues involving social, political, and behavioural factors. Second, the dataset is suited to advanced statistical methods that can explore heterogeneity in the uptake of behavioural health policies, such as vaccine nudges. Third, the timing of the data collection, coinciding with the rise of the Omicron variant, provides valuable insights into why some previously vaccinated individuals might hesitate to receive additional doses, potentially improving our understanding of the COVID-19 pandemic and possible responses to pandemics and other public health emergencies in the future.

## Introduction

Early 2022 saw a new phase of the COVID-19 pandemic. It was 2 years on from the first international policy measures that were implemented to contain the virus. Publics in many countries had been through multiple ‘lockdowns’ while vaccines were developed at unprecedented speed. The launch and rollout of COVID-19 vaccines in late 2020 and early 2021 promised change, but the pandemic continued to shape work, social, and family life. There was varied but generally high take-up of initial COVID-19 vaccines across the G-7, and to a lesser extent the booster vaccines offered ahead of the 2021/22 winter season.

A key public policy challenge at the time was how to maintain vaccine uptake when facing public resistance to vaccination and declining willingness to engage in protective measures more generally. This challenge motivated an investigation of strategies for citizen engagement to understand when and how they might affect vaccine take-up.

Vaccine promotion policies in the G-7 countries, much like wider COVID-19 public health policies, ranged from more to less stringent (so called ‘hard’ and ‘soft’ policies; see Banerjee et al., 2021, 2023) based on the relative costs of compliance and non-compliance. More stringent policies included vaccine mandates, travel passes requiring proof of vaccination, and fines; while less stringent policies included information campaigns and behavioural nudges like text message reminders, automatic appointments, and social norm appeals. Behavioural nudges are tools informed by psychological insights, which are liberty preserving, cost-effective, and easy to administer (Thaler & Sunstein, 2008). They promote behaviour change through encouragement and persuasion rather than rules and financial incentives, so were a potential alternative to more stringent policies. While part of the appeal of nudges is that they preserve personal liberties and freedom of choice, they present different risks such as reduced vaccination coverage and public health benefits since nudges are often easy to ignore.

Our survey was fielded at a time when there was growing interest in identifying less stringent approaches to managing the pandemic, partly in recognition that the unprecedented policies (such as the EU COVID-19 pass, travel restrictions, widespread school and business closures, and vaccine mandates for health workers) implemented during 2021 were unlikely to be sustainable (see, for example, protests in early 2022 in Canada and France). Behavioural scientists hoped that insights from their research would encourage higher vaccine uptake while minimising restrictions and reducing backlash (Bavel et al., 2020).

Against this context, we set out to measure public attitudes towards COVID-19 vaccines, vaccine take-up, and public support for vaccine policies across the G-7.^1^ Investigating support for policies is important in liberal democracies where the legitimacy of governmental action relies on public acceptance of policy approach (Capano & Lippi, 2017). It also allows us to relate our work not only to broader scholarship on public attitudes for government action but also preferences for particular policy approaches (Sunstein et al., 2018), and how policy support can be affected by competing concerns for policy effectiveness and personal liberties. The dataset enables analysis of attitudes towards vaccines and policies that promote vaccines, including through comparisons with subsequent data collection efforts to assess if and how attitudes change over time. In this way, our dataset offers insights into future health crises as well as efforts to promote immunization against common infections such as flu and measles.

## Materials and methods

The study was approved by the Ethical Review Board of King’s College London (reference: MRA-21/22-26861). The data collected in available on OSF (https://doi.org/10.17605/OSF.IO/6MKWG). The pre-registration plan is available online here: Appendix 1 provides details on our experimental vignettes and outcome variables and Appendix 2 outlines the codebook for interoperability of use of our dataset.

### Data collection

We fielded two surveys online to a total of 42,417 participants in 2022: wave 1 was fielded from January 27 to February 26 (*n* = 24,303) and wave 2 from March 6 to May 12 (*n* = 18,114). Participants were recruited by the survey research company Dynata, and each wave had its own pre-testing and a pilot phase. This involved running the survey with a small sample of the target population to ensure the survey instrument was coherent, and then a soft launch to check and correct procedural flows. Sample size was informed by ex-ante power analysis which was pre-registered before each wave. Respondents were compensated based on Dynata’s standard survey incentives, which may have involved cash or points. The survey was written in English and professionally translated into French, German, Italian, and Japanese. Written, informed, and explicit consent was required from all participants who chose to participate in the study.

Broadly speaking, responses were evenly spread across the seven countries (see Table 1). Sample sizes were based on an ex-ante power analysis that was pre-registered on Open Science Framework (see https://doi.org/10.17605/OSF.IO/6MKWG). Quotas were applied to deliver country samples representative of age, gender, educational background and subnational region (with surplus responses dropped).

**Table 1.** Dataset by country and wave.

	Wave 1	Wave 2	Total *N*	%
Canada	3470	2566	6036	14.2
France	3485	2623	6108	14.4
Germany	3485	2568	6053	14.3
Italy	3487	2565	6052	14.3
Japan	3421	2674	6095	14.4
UK	3472	2552	6024	14.2
USA	3483	2566	6049	14.3
Total	24,303	18,114	42,417	100

### Survey design

The survey instrument was designed using Qualtrics. The survey contained three experiments that sought to (a) understand public attitudes towards vaccination promotion interventions and (b) determine whether it was possible to deploy interventions that respect personal autonomy but persuade people to get vaccinated. In wave 1 of the survey, the first and third experiments were designed to assess the effectiveness of a new modified toolkit of behavioural science and public policy – nudge+ (Banerjee & John, 2024). Nudge+, which encourages people to reflect on the nudge at the time of its delivery, was previously shown to deliver more effective and legitimate pro-environmental behaviours (Banerjee et al., 2023). The current study allowed us to test whether it could conceivably increase public support for, and effectiveness of, nudges in the context of vaccinations.

The first experiment assessed the effects of different types of policy attributes on support for COVID-19 policies. In this conjoint experiment, respondents were randomly shown three pairs of vaccine policies that contain four independently randomised attributes. The first two attributes of appointment scheduling and sending reminders contained values that corresponded to classic nudge policies (default scheduling/automatic reminders) as well as those that include a nudge+ element (calling to discuss questions about COVID-19 vaccines before scheduling an appointment or sending a reminder). We also tested the effect of employer mandates and government fines on COVID-19 policy support. Results from this study are currenty being written up.

The second experiment evaluated the relationship between perceived vaccine effectiveness and support for more stringent vaccine promotion policies (see Koenig et al., 2024). In this between-subjects experiment, we randomly varied the effectiveness of a hypothetical COVID-19 booster vaccine against a new variant. Vaccine effectiveness levels were set at 50%, 60%, 70%, 80%, and 90%. We measured support for several policy measures that varied from lower to higher stringency. A higher stringency measure includes a vaccine mandate that employers could enforce, while a lower stringency measure includes promoting the vaccine with advertisements. These measures were developed using real world examples, and are set out in full in the supplementary materials (see Appendix 1).

Finally, the third experiment presented respondents with another future COVID-19 policy scenario and randomised people into one of four conditions in a between-subjects design (see Banerjee et al., 2024b). This experiment was designed to test the role of reflection in nudges in improving effectiveness and legitimacy of behavioural nudges, especially when combined, an approach that has been labelled as nudge+ as outlined earlier (Banerjee & John, 2024). It has been suggested that reflective approaches can help empower citizens by building more autonomy and agency (Banerjee et al., 2024a). The four treatment cobditions are summarized below

- Control group: The government leaves vaccine choice up to individuals and requires them to call to get an appointment to get a booster.
- Nudge group: Every adult will be automatically enrolled to receive a booster shot at a local clinic that will call them to schedule a booster appointment at a convenient date and time.
- Think group: Respondents are provided the control group prompt and then are asked to think about the government's actions in this scenario, whether this approach is appropriate, and whether this approach will work for them, writing their thoughts down in a text box.
- Nudge+: Respondents are provided the nudge group prompt, and then are asked to think about the government's actions in this scenario, whether this approach is appropriate, and whether this approach will work for them, writing their thoughts down in a text box.

We measured support for the policy and willingness to be vaccinated in each of these four experimental conditions. Outcome measures aimed to delve into specific research questions around trade-offs between policy effectiveness and legitimacy (acceptability) of behavioural nudges. We designed bespoke outcome measures to suit our study aims.

**Figure 1.**
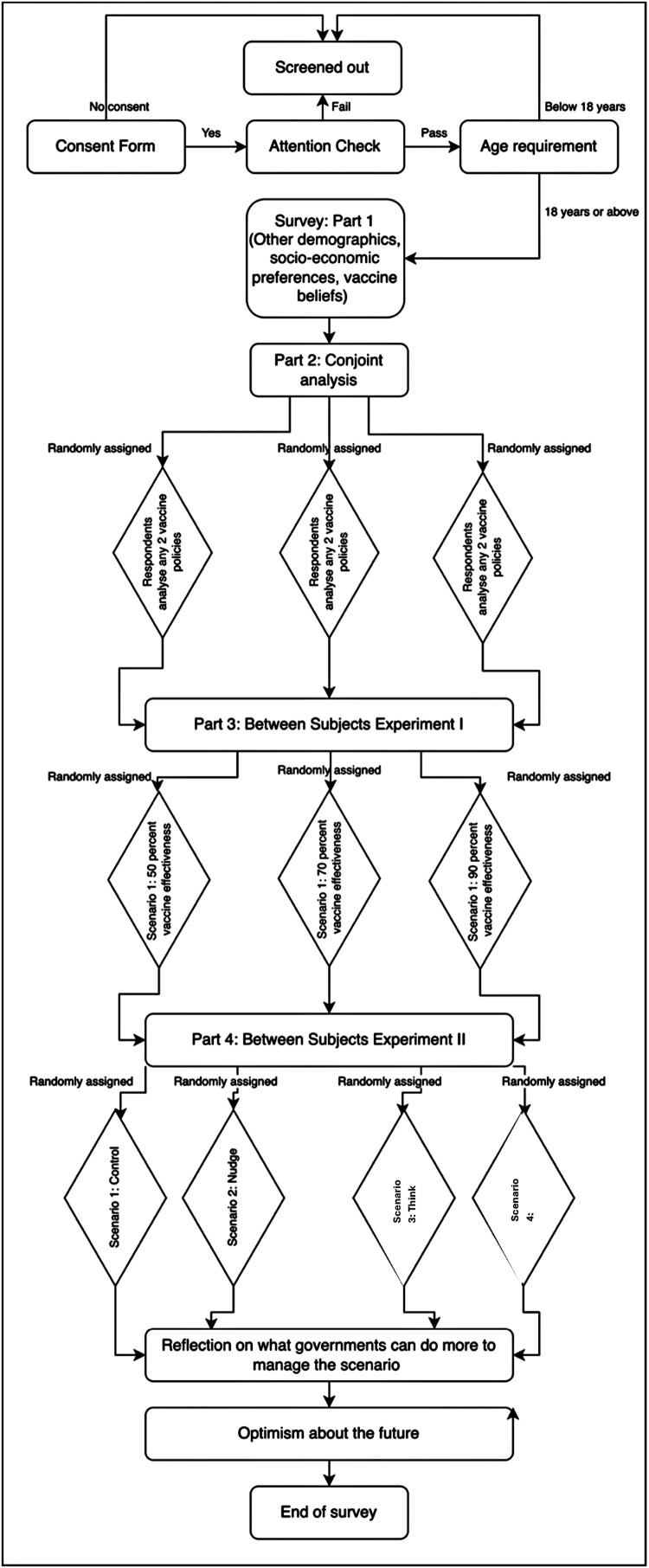
Survey flow of wave 1. The survey flow is described in the figure. Participants who did not consent or failed the attention check or did not meet the age requirement were screened out from the survey. Eligible participants continued to report socio-demographic characteristics. Then they were entered into the conjoint experiment, following which they were entered into two different between-subjects’ experiment. The first between-subjects assessment evaluated people’s support for a vaccine policy given varying levels of vaccine efficacy whereas the second experiment assessed people’s intentions for taking up booster vaccination and its support based on four randomised policy scenarios. Participants reported optimism about the future before ending the survey.

Alongside these experiments, we collected a diverse range of pre-treatment covariates as well as basic socio-demographic characteristics of the respondents, many of which were tried and tested survey questions from prior research and featured in prior research examining factors that affected vaccine take-up intentions and attitudes. To improve the quality of survey responses, we followed several state-of-the-art procedures. For example, we included multiple attention screeners Berinsky et al. (2021) to prevent inattentive responses and minimise speeders. We implemented a technique to exclude VPN users located outside the country in which the survey was fielded.^2^ Incomplete surveys were dropped, as were repeat surveys taken by the same Dynata ID. A further 27 observations were dropped (wave 1) due to some respondents being unable to load the Javascript programming. Figure 1 visualises the survey flow.

Survey wave 2 was fielded after preliminary data analysis of wave 1 data. This provided an opportunity to modify the survey flow and some content of the experiments. These changes mainly affected the ordering of the three experiments and the precise wording of experimental vignettes, not the causal objectives of the experiments. For example, in wave 2, the nudge plus experiment preceded the conjoint, which preceded the policy stringency experiment. This modification was made to minimise the possibility of carryover effects from the future scenario experiment. We added four new treatment arms to the nudge+ experiment, diversifying the vignettes. In the policy stringency experiment, we varied the levels of vaccine effectiveness in relative terms (more/less/the same as the previous vaccine) instead of percentage terms and added additional policy measures to expand our index of support for stringent policy. Finally, we modified the attributes and levels of the conjoint experiment to simplify their framing for respondents. These changes were made transparently and pre-registered prior to the launch of the second wave (for details, see https://doi.org/10.17605/OSF.IO/6MKWG). English versions of both survey waves are available in Appendix 3. All translated versions of the surveys are available online via OSF.

## Data descriptions

A selected list of variables and descriptive statistics is set out in Table 2. The dataset offers the advantage of further granularity; for example, each of these variables can be explored at the level of country and subnational regions. For our current purposes we report means for answers choosing a substantive response option.

**Table 2.** Selected descriptive statistics.

Variable	Mean (%)	Standard deviation	Number of observations (*n*)
***Demographics***			
Female (0 for man, another gender, or non-binary/1 for female)	51.6%	–	42,374
Age	47.2 years	16.4	42,417
Parent (0/1)	0.49	0.50	42,381
Education (0/1 for college degree)	0.29	0.5	42,417
Where do you live?	–	–	42,400
Rural area	9.8%	–	4148
Town	17.7%	–	7506
Small city	20.3%	–	8611
Suburb near large city	25.1%	–	10,620
Large city	27.2%	–	11,515
How important is religion in your life?	–	–	42,393
Not at all	30.4%	–	12,892
Not very	26.0%	–	11,016
Somewhat	26.8%	–	11,342
Very	16.9%	–	7143
***Social***			
How much do you agree with ‘politicians usually ignore my community’ (0–10 scale of strongly disagree to strongly agree)	6.23	2.6	42,149
How much do you agree ‘saving lives is more important than personal liberty’ (0–10 scale of strongly disagree to strongly agree)	7.06	2.48	42,078
How often have you read, listened to, or watched news related to the COVID-19 pandemic over the past week?	–	–	42,360
Never	8.0%	–	3379
Once	5.6%	–	2391
A few times	18.3%	–	7768
Almost every day	18.2%	–	7723
Daily	28.9%	–	12,231
Several times a day	20.9%	–	8868
***Health and COVID-19***			
Have you been infected with COVID-19 since the start of the pandemic? (0/1)	21.5%	–	42,376
Have you received a COVID-19 vaccine?	–	–	42,417
Yes a one-shot vaccine	8.7%	–	3678
Yes first dose of a two-shot vaccine	6.4%	–	2702
Yes two doses of a two-shot vaccine	71.7%	–	30,404
No vaccine	13.2%	–	5585
How much do you agree ‘without a vaccine I am likely to catch COVID-19’? (0–10 scale of strongly disagree to strongly agree)	6.35	3.1	42,170
Can COVID-19 vaccines be trusted? (0/1)	–	–	42,138
Yes	78.0	–	32,997
No	22.0	–	9321
How frequently do you engage in ‘avoiding small indoor gatherings less than 6 people’? (0–10 scale of never to always)	5.74	3.3	40,167
How frequently do you ‘avoid going to pubs/bars/restaurants?’ (0–10 scale of never to always)	5.93	3.3	38,855
***Political***			
Strength of partisan identity (very strongly / fairly strongly / not very strongly / don’t know)			29,382
Very strongly	23.9%	–	7015
Not very strongly	22.8%	–	6685
Fairly strongly	51.3%	–	15,064
Don’t know	2.1%	–	618
Left–right politics scale (0–10 scale of left to right)	5.27	2.3	41,452
Confidence in political institutions: Government (0–10 scale of no confidence at all to great deal of confidence)	4.31	2.8	42,053
How strongly do you agree ‘what people call compromise in politics is really just selling out on one’s principles’ (1–5 scale of strongly disagree to strongly agree)	–	–	42,345
Strongly disagree	3.3%	–	1396
Somewhat disagree	11.0%	–	4661
Neither agree nor disagree	35.1%	–	14,861
Somewhat agree	32.1%	–	13,592
Strongly agree	18.5%	–	7853
***Psychological***			
Willingness to take risks (0–10 scale of completely unwilling to very willing)	5.16	2.7	42,114

*Notes*: The variable for ‘female’ is created from the original survey question on gender, where response options were man/woman/non-binary/another/prefer not to say.

We created a binary variable for female based on selecting the response option ‘woman’ to assess randomisation balance (as part of our pre-registered approach). The variable for education is created from the original survey question on education, which offered a range of options for the highest level of education completed from primary school to university. These options varied for local context. For our research purposes, we created a binary variable based on whether the equivalent of a university undergraduate degree had been selected. Percentages reported above are rounded up so may not add to 100%. Variables have different numbers of observations due to missing responses. Skipped responses are considered missing data, and response options comprising ‘don’t know’ and ‘prefer not to answer’ are not included in the table above, but are available in the dataset.

## Discussion

The data presents opportunities to explore associations and relationships between vaccine attitudes and a host of factors including individual characteristics, political attitudes, risk preferences, and media consumption. The focus on G-7 nations allow for comparative analysis of countries who share liberal democratic values, income levels, health system capabilities, a very similar timeframe in terms of when vaccines were available and rolled out as part of pandemic containment policies, and a number of similar public health and policy responses to the pandemic. The data can also be compared to subsequent data collection to investigate changes over time. We provide three illustrative examples below to demonstrate the variety of research investigations that could be undertaken with the dataset: (1) trust in vaccines and political beliefs, (2) factors associated with public health behaviours, and (3) social media consumption and COVID-19 attitudes.

### Trust in vaccines by political beliefs

Our dataset suggests that respondents who state they do not trust the COVID-19 vaccine also report more conservative attitudes on a seven-point left–right scale where higher values are more conservative (see Table 3). These differences are relatively small – for example, the sample average was 5.6 points on the left–right scale for those who do not trust the vaccine compared to 5.2 points for those who do – but statistically significant. This relationship holds for each of the country samples except for France and the UK, where the difference in ideology is not statistically significant at the 5% level. The difference in left–right preferences between those who do and do not trust the vaccine is largest in the USA sample.

**Table 3.** Political attitudes and trust in the vaccine.

Politics left–right mean score (SE)	Does not trust vaccine (1)	Trusts vaccine (2)	*P*-value on hypothesis test (1) = (2)
Whole sample (*n* = 41,411)	5.61 (0.02)	5.18 (0.01)	0.000
Canada (*n* = 5,902)	5.76 (0.07)	5.46 (0.03)	0.000
France (*n* = 6,002)	5.52 (0.06)	5.40 (0.04)	0.084
Germany (*n* = 5,978)	5.23 (0.05)	4.65 (0.03)	0.000
Italy (*n* = 5,940)	5.86 (0.08)	4.80 (0.04)	0.000
Japan (*n* = 5,650)	5.11 (0.05)	5.28 (0.02)	0.002
UK (*n* = 5,956)	5.25 (0.07)	5.30 (0.03)	0.581
USA (*n* = 5,983)	6.34 (0.06)	5.40 (0.04)	0.000

*Notes*: Standard errors in parentheses below mean value of responses using politics left–right scale, where 0 is most left and 10 is most right.

### Factors associated with public health behaviours

We include a battery of questions that ask about compliance with various public health guidance and rules that were in place during the pandemic, such as avoiding bars and restaurants, avoiding large indoor gatherings, and wearing face masks in public places. Unsurprisingly, older groups tended to report they were more likely to follow such rules than younger groups; for example, the over-65s reported an average score of 7.5 when asked whether they avoid large indoor gatherings versus 6.2 for respondents aged 18–24 (on a scale of 0–10, where 0 is never avoid and 10 is always avoid).

**Figure 2.**
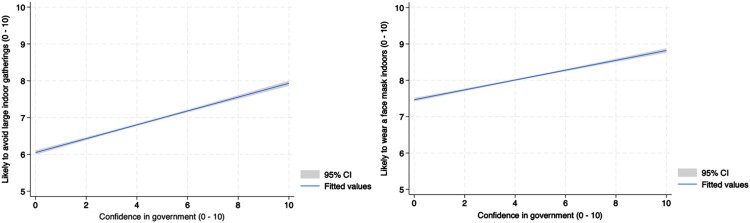
(left) Confidence in government and likelihood to avoid large indoor gatherings; (right) Confidence in government and likelihood to wear a face mask indoors. The left panel of figure shows a positive correlation between individuals’ confidence in government and likelihood to avoid large indoor gatherings whereas the right panel of figure shows a positive correlation between individuals’ confidence in government and likelihood to wear a face mask indoors.

In addition, our data can be used to answer questions about how and whether confidence in public institutions is associated with the likelihood of avoiding public health risks. For example, where greater confidence in government is reported, respondents are more likely to avoid large indoor gatherings and more likely to wear a face mask indoors (see Figure 2).

### Social media consumption and COVID-19 attitudes

**Figure 3.**
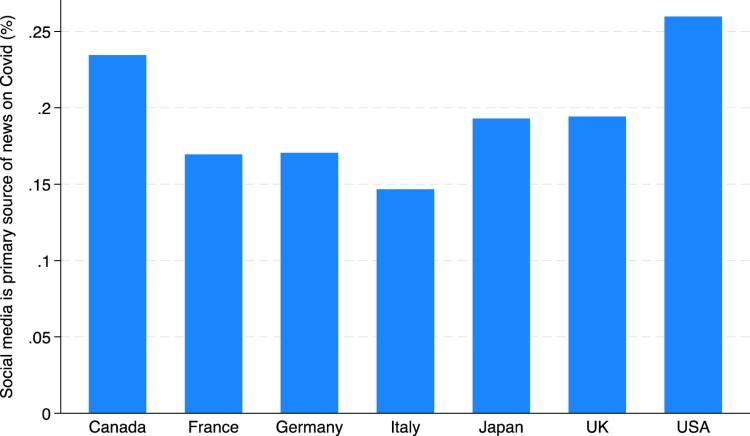
Social media is primary news source (%) by country. Figure plots the proportion that social media is the primary source of news on COVID-19 across the G-7 countries. The proportion is the highest in the USA (slightly more than 25%) and lowest in Italy (slightly below 15%).

Respondents drew on a range of news sources to learn about COVID-19, including newspapers (including websites), radio, TV, and social media platforms such as WhatsApp and WeChat. Social media was the primary news source for a sizeable minority of respondents: almost 20% across the whole sample, with notable variation across countries as illustrated in Figure 3.

**Figure 4.**
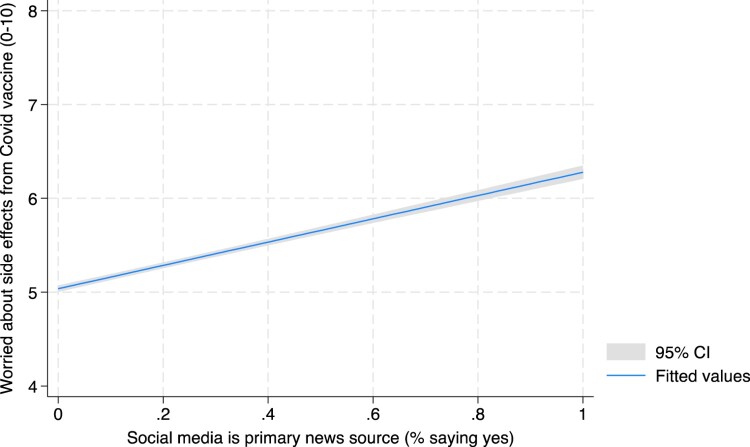
Social media as the primary news source predicts higher levels of concern about COVID-19 vaccine side effects. Figure shows a positive correlation between social media as the primary news source and higher levels of concern about COVID-19 vaccine side effects in the pooled sample of G-7 countries.

Our dataset can be used to investigate how social media consumption relates to a variety of other outcomes including trust in the vaccine, vaccine status, and concerns about potential side effects. For example, respondents who cite social media as their primary news source are significantly less likely to say they trust the vaccine (only 64% compared to 81% of those who mention traditional media as their primary Cov news source). This opens up possibilities for users to investigate whether and how social media is associated with other attitudes towards the vaccines. A brief illustration is set out in Table 4 and Figure 4.

**Table 4.** Social media consumption and attitudes towards COVID-19.

Attitudes Agreement with statement on scale of 0–10	Social media as primary news source (1)	Traditional media as primary news source (2)	*P*-value on hypothesis test (1) = (2)
I am worried about potential side effects from the COVID-19 vaccine	6.28 (0.03)	5.04 (0.02)	0.000
Too much fuss is being made about the risk of COVID-19	5.91 (0.08)	4.37 (0.05)	0.000

We acknowledge certain limitations of our dataset, such as its bespoke experimental design, and bespoke outcome measures may reduce comparability with outcome measures used in other studies. Despite the cross-country nature of data collection, this may not be fully comparable with countries having different political and economic regimes, or from the Global South. We also point out the hypothetical nature of our outcomes meaning we can point to behavioural intentions and not outcomes. Another limitation is categorisation of data (such as gender into a binary variable) which can lead to loss of granularity at the expense of more information for ease of data analysis as pre-registered (such as for joint tests for randomisation). Future research should use robustness checks to explore this granular information. We have included a detailed codebook in Appendix 2 for interoperability and reusability of the data following FAIR principles.

Future research designs could usefully draw upon the survey instrument, observational data, and experimental designs embedded within the survey to support the design of future studies and to support broader efforts for replication and generalisability. While we present straightforward bivariate associations above for illustrative purposes, the dataset lends itself well to multivariate analysis and more sophisticated modelling using multi-level regressions, heterogeneity and sub-group analysis, in-depth analysis of specific countries, and time series analysis.

Our dataset adds to a growing body of data on public attitudes and beliefs towards COVID-19 vaccinations, and complements existing datasets. For example, with the COVIDiSTRESS dataset, Blackburn and Vestergren (2022) present global data on psychological and behavioural outcomes 1 year in to the pandemic; Klumpp et al. (2022) have a tighter regional focus with public opinion data from Germany and the USA; and Cheng et al. (2024) concentrate on harmonising eight other datasets that report on public health and social measures prior to September 2021. We offer a unique combination of social, political and behavioural variables, collected as experimental and observation data, at an important midway point of the pandemic.

## Supplementary Material

Data Paper Appendix 1.docx

Data Paper Appendix 3.docx

Data Paper Appendix 2.docx

## Data Availability

Data is available in Open Science Framework (see: 10.17605/OSF.IO/6MKWG). Appendix 2 provides readers with a user-friendly codebook to understand how data was collected, cleaned and stored.

## Notes

1 Data collection was funded by the British Academy under the ‘COVID-19 Recovery: Building Future Pandemic Preparedness and Understanding Citizen Engagement in the USA and UK’ grant scheme. We included the other five countries in the G-7 as they provided a valuable, comparable set of cases, being liberal democracies and advanced economies.

2 We used simple branch logic in Qualtrics to remove respondents who did not load the required Javascript. Using this method, we rejected 48 (0.16%) potential non-target respondents from attempting our second survey.
