# Supplementary material for: What nudges you to take a vaccine? Understanding behavioural drivers of COVID-19 vaccinations using large-scale experiments in the G-7 countries
Source: Health Psychol Behav Med. 2025 Apr 16;13(1):2490550. doi: 10.1080/21642850.2025.2490550 (PMC12004716; doi:10.1080/21642850.2025.2490550)
Supplement: Data Paper Appendix 1.docx [file RHPB_A_2490550_SM5917.docx]

Appendix 1

***From anonymised citation***

**Study 1 vignette attributes, and dependent variable**

Imagine the following scenario:

In October of 2022, a new variant emerges which, like Omicron, is highly contagious. New vaccine boosters are developed.

These boosters provide **[50% / 60% / 70% / 80% / 90%]** protections against infection from the new variant.

The government in your country would like individuals to take this booster shot.

Which of the following policies would you support?

**Policy 1:** No one should be forced to take the booster but it should be available to anyone who wants it.

**Policy 2:** Those who do not take the booster should be stopped from entering any indoor public spaces (e.g. restaurants, entertainment venues) and/or using public transport.

**Policy 3:** Employers should require their employees to get the booster.

**Policy 4:** Those who do not take the booster should be fined by the government.

Response options for each policy attribute: **Yes / No / Unsure**

**Study 2 vignette, attributes, and dependent variable**

Next we’d like you to imagine a new hypothetical scenario:

A new and highly contagious variant of COVID-19 emerges several months from now. A new booster has been developed to combat this new variant.

This new booster is safe and recommended for everyone regardless of whether they had previously had any COVID-19 vaccinations.

This new booster is **[less / as / more]** effective at preventing COVID-19 infection than previous vaccines.

The government would like people to get this new booster.

Which of the following policies would you support?

**Policy 1:** The government makes this new booster freely available to all eligible adults.

**Policy 2:** The government promotes this new booster using advertisements.

**Policy 3:** The government sends text messages to eligible adults reminding them that this new booster is available.

**Policy 4:** The government gives a tax break to eligible adults who get this new booster.

**Policy 5:** The government allows employers to require their eligible employees to get this new booster.

**Policy 6:**The government requires eligible adults to show proof that they got this new booster before they can enter certain indoor places such as restaurants, gyms and theatres.

**Policy 7:** The government fines eligible adults who refuse this new booster.

Response options for each policy attribute: **I would support / I would oppose**
