# Supplementary material for: What nudges you to take a vaccine? Understanding behavioural drivers of COVID-19 vaccinations using large-scale experiments in the G-7 countries
Source: Health Psychol Behav Med. 2025 Apr 16;13(1):2490550. doi: 10.1080/21642850.2025.2490550 (PMC12004716; doi:10.1080/21642850.2025.2490550)
Supplement: Data Paper Appendix 3.docx [file RHPB_A_2490550_SM5916.docx]

Appendix 3

UK-Wave 1

Survey Flow

EmbeddedData

psidValue will be set from Panel or URL.

Standard: ethics (2 Questions)

Branch: New Branch

If

If Thank you for taking the time to participate in this study.    Please note that you need to be 18... I DO NOT give my consent to participate in this research study. Is Selected

EndSurvey: Advanced

Branch: New Branch

If

If What is your age? Please enter as a number (e.g., 25). Text Response Is Less Than or Equal to 17

EndSurvey: Advanced

Standard: captcha (1 Question)

Standard: Screens1 (1 Question)

Branch: New Branch

If

If Help us keep track of who is paying attention - please select “somewhat disagree” in the options... Somewhat disagree Is Not Selected

EndSurvey: Advanced

Standard: Demographics (13 Questions)

Branch: New Branch

If

If Do you currently live in the United Kingdom? No Is Selected

EndSurvey: Advanced

Standard: Party ID (3 Questions)

Standard: Scales (5 Questions)

Standard: covid19 (16 Questions)

Standard: Media consumption (2 Questions)

Standard: Screen2 (1 Question)

Standard: conjoint (19 Questions)

Standard: screens3 (1 Question)

BlockRandomizer: 1 -

Standard: treatment1 (9 Questions)

Standard: treatment2 (9 Questions)

Standard: treatment3 (10 Questions)

Standard: treatment4 (10 Questions)

Standard: post-treatment (8 Questions)

EmbeddedData

effectiveValue will be set from Panel or URL.

scheduleValue will be set from Panel or URL.

remindersValue will be set from Panel or URL.

mandateValue will be set from Panel or URL.

finesValue will be set from Panel or URL.

schedule1Value will be set from Panel or URL.

reminders1Value will be set from Panel or URL.

mandate1Value will be set from Panel or URL.

fines1Value will be set from Panel or URL.

schedule2Value will be set from Panel or URL.

reminders2Value will be set from Panel or URL.

mandate2Value will be set from Panel or URL.

fines2Value will be set from Panel or URL.

schedule3Value will be set from Panel or URL.

reminders3Value will be set from Panel or URL.

mandate3Value will be set from Panel or URL.

fines3Value will be set from Panel or URL.

schedule4Value will be set from Panel or URL.

reminders4Value will be set from Panel or URL.

mandate4Value will be set from Panel or URL.

fines4Value will be set from Panel or URL.

schedule5Value will be set from Panel or URL.

reminders5Value will be set from Panel or URL.

mandate5Value will be set from Panel or URL.

fines5Value will be set from Panel or URL.

Q_TerminateFlagValue will be set from Panel or URL.

EndSurvey: Advanced

| Page Break |  |
| --- | --- |

Start of Block: ethics

| 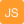 |
| --- |

ethics
 Thank you for taking the time to participate in this study.    Please note that you need to be 18+ and speak English fluently, otherwise please don't partake. The study should take around 15 minutes to complete. In the study, you will be asked a series of questions about vaccines. You will be paid for your participation in the survey. Just make sure to read all the instructions carefully and try your best.    NOTE: You can withdraw from the study at any stage without providing an explanation. Your privacy is very important, so we always use anonymised data. Results from this work may be written up for publication in a peer reviewed journal. However, individual data will never be published, and we will not hold personal identifiers. This project is in line with the ethical guidelines established by the Research Ethics Committee of King’s College London.   For more details about this research project, please see this information sheet.
 
If you have any questions you would like to ask before starting the survey, please feel free to contact Professor Peter John, King’s College London: peter.john@kcl.ac.uk. 
 
Please note in this survey we would like to ask some questions that may be perceived as sensitive, such as gender, ethnicity, political orientation, and religion. Providing information in response to these questions is entirely voluntary and you may withdraw your consent at any time. If you are happy to participate, please choose "I give my consent to participate in this research study."

- I DO NOT give my consent to participate in this research study. (1)
- I give my consent to participate in this research study. (2)

Skip To: End of Block If Thank you for taking the time to participate in this study.    Please note that you need to be 18... = I DO NOT give my consent to participate in this research study.

| Page Break |  |
| --- | --- |

| 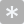 |
| --- |

yob What is your age? Please enter as a number (e.g., 25).

________________________________________________________________

Skip To: End of Block If Condition: What is your age? Please en... Is Less Than or Equal to 17. Skip To: End of Block.

End of Block: ethics

Start of Block: captcha

captcha Before you proceed to the survey, please complete the Captcha below.

End of Block: captcha

Start of Block: Screens1

screen1 Help us keep track of who is paying attention - please select “somewhat disagree” in the options below.

- Strongly disagree (1)
- Somewhat disagree (2)
- Neither agree nor disagree (3)
- Somewhat agree (4)
- Strongly agree (5)

Skip To: End of Block If Help us keep track of who is paying attention - please select “somewhat disagree” in the options... = Strongly disagree

Skip To: End of Block If Help us keep track of who is paying attention - please select “somewhat disagree” in the options... = Neither agree nor disagree

Skip To: End of Block If Help us keep track of who is paying attention - please select “somewhat disagree” in the options... = Somewhat agree

Skip To: End of Block If Help us keep track of who is paying attention - please select “somewhat disagree” in the options... = Strongly agree

End of Block: Screens1

Start of Block: Demographics

country Do you currently live in the United Kingdom?

- Yes (1)
- No (2)

Skip To: End of Block If Do you currently live in the United Kingdom? = No

| Page Break |  |
| --- | --- |

subnat_region Which region do you currently live in?

- East Anglia (1)
- East Midlands (3)
- London (4)
- North East (5)
- North West (6)
- Northern Ireland (7)
- Scotland (8)
- South East (9)
- South West (10)
- Wales (11)
- West Midlands (12)
- Yorkshire & Humberside (13)
- Prefer not to answer (2)

| Page Break |  |
| --- | --- |

gender Are you...

- A man (1)
- A woman (2)
- Non-binary (3)
- Another gender (please specify) (4) __________________________________________________
- Prefer not to answer (5)

| Page Break |  |
| --- | --- |

education What is the highest level of education you have achieved?

- Combined Junior and Infant School/ Infant School (1)
- Junior School (11)
- Comprehensive School (12)
- Comprehensive School (GCSE)/ Secondary Modern (GCSE)/ Grammar School (GCSE)/ City Technology College (GCSE)/ Sixth Form (13)
- College/ Institution of Higher education (14)
- Open College - College of Technology - Institute/ Teacher Training College (15)
- University/ Open University (16)
- Prefer not to answer (2)

| Page Break |  |
| --- | --- |

citizen Are you a citizen of the United Kingdom?

- Yes (1)
- No (2)

| Page Break |  |
| --- | --- |

parent_screen Are you a parent or guardian to any children?

- Yes (1)
- No (2)

| Page Break |  |
| --- | --- |

Display this question:

If Are you a parent or guardian to any children? = Yes

| 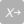 |
| --- |

children How many children under the age of 18 are you the parent or guardian of?

▼ 0 (4) ... 20 or more (24)

| Page Break |  |
| --- | --- |

| 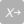 |
| --- |

children_u18 Are there any children under the age of 18 living in your household?

▼ 0 (1) ... 20 or more (21)

| Page Break |  |
| --- | --- |

ethnicity Which of the following best describes your ethnicity?

- White: British (1)
- White: Irish (2)
- White: Other (3)
- Mixed: White and Black Caribbean (4)
- Mixed: White and Black African (5)
- Mixed: White and Asian (6)
- Mixed: Other mixed background (7)
- Black or Black British: African (8)
- Black or Black British: Caribbean (9)
- Black or Black British: Any other Black background (10)
- Asian or Asian British: Indian (11)
- Asian or Asian British: Pakistani (12)
- Asian or Asian British: Bangladeshi (13)
- Asian or Asian British: Other Asian background (14)
- Chinese (15)
- Other ethnic group not represented by these options (please specify) (16) __________________________________________________
- Do not wish to say (17)

| Page Break |  |
| --- | --- |

employment What is your employment status? Please select as many as applicable.

- Working for pay full-time (1)
- Working for pay part-time (2)
- Self-employed (3)
- Retired (4)
- Unemployed / Looking for work (5)
- Student (6)
- Caring for family (7)
- Other (Please specify) (8) __________________________________________________

| Page Break |  |
| --- | --- |

income What was your total household income, before taxes, for the year 2021?

- No income (1)
- £1 - £4,400 (2)
- £4,401 - £8,800 (4)
- £8,801 - £17,600 (5)
- £17,601 - £26,400 (6)
- £26,401 - £35,200 (7)
- £35,201 - £52,800 (8)
- £52,801 - £64,500 (9)
- £64,501 - £88,000 (10)
- £88,001 - £117,300 (11)
- More than £117,300 (12)
- Don't know / prefer not to answer (3)

| Page Break |  |
| --- | --- |

urban_rural Which of the following best describes the place where you now live…

- A large city (1)
- A suburb near a large city (2)
- A small city (3)
- A town (4)
- A rural area (5)

| Page Break |  |
| --- | --- |

religiosity In your life, you would say religion is:

- Very important (1)
- Somewhat important (2)
- Not very important (3)
- Not at all important (4)

End of Block: Demographics

Start of Block: Party ID

| 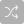 | 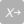 |
| --- | --- |

partyid In politics, do you usually think of yourself as a(n):

- Conservative Party (1)
- Labour Party (2)
- Scottish National Party (3)
- Liberal Democrats (4)
- Democratic Unionist Party (5)
- Sinn Féin (6)
- Plaid Cymru (7)
- Social Democratic and Labour Party (8)
- Green Party (9)
- Alliance Party of Northern Ireland (10)
- Another party (specify): (11) __________________________________________________
- No party (12)
- Don't know (13)

| Page Break |  |
| --- | --- |

Display this question:

If In politics, do you usually think of yourself as a(n): = Conservative Party

Or In politics, do you usually think of yourself as a(n): = Labour Party

Or In politics, do you usually think of yourself as a(n): = Scottish National Party

Or In politics, do you usually think of yourself as a(n): = Liberal Democrats

Or In politics, do you usually think of yourself as a(n): = Democratic Unionist Party

Or In politics, do you usually think of yourself as a(n): = Sinn Féin

Or In politics, do you usually think of yourself as a(n): = Plaid Cymru

Or In politics, do you usually think of yourself as a(n): = Social Democratic and Labour Party

Or In politics, do you usually think of yourself as a(n): = Green Party

Or In politics, do you usually think of yourself as a(n): = Alliance Party of Northern Ireland

partyid_strength How strongly ${partyid/ChoiceGroup/SelectedChoices} do you feel?

- Very strongly (1)
- Fairly strongly (2)
- Not very strongly (3)
- Don't know (4)

| Page Break |  |
| --- | --- |

Display this question:

If In politics, do you usually think of yourself as a(n): = Another party (specify):

And And In politics, do you usually think of yourself as a(n): Text Response Is Not Empty

partyid_strength How strongly ${partyid/ChoiceTextEntryValue/2} do you feel?

- Very strongly (1)
- Fairly strongly (2)
- Not very strongly (3)
- Don't know (4)

End of Block: Party ID

Start of Block: Scales

risk On a scale of 0-10, where **0** means you are ‘**completely unwilling to take risks’** and **10** means you are ‘**very willing to take risks’** in general, how willing or unwilling are you to take risks?

|  | Completely unwilling to take risks | Very willing to take risks |
| --- | --- | --- |

|  | 0 | 1 | 2 | 3 | 4 | 5 | 6 | 7 | 8 | 9 | 10 |
| --- | --- | --- | --- | --- | --- | --- | --- | --- | --- | --- | --- |

| Page Break |  |
| --- | --- |

lr_scale In political matters, people talk of the '**left**' and the '**right**'. How would you place your views on this scale, generally speaking?

|  | Left | Right |
| --- | --- | --- |

|  | 0 | 1 | 2 | 3 | 4 | 5 | 6 | 7 | 8 | 9 | 10 |
| --- | --- | --- | --- | --- | --- | --- | --- | --- | --- | --- | --- |

| 1 () | 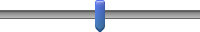 |
| --- | --- |

| Page Break |  |
| --- | --- |

pol_system_t Timing

First Click (1)

Last Click (2)

Page Submit (3)

Click Count (4)

| 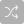 |
| --- |

pol_system On a scale of 0-10, where **0** means you have ‘**no confidence at all**’ and a **10** means you have a ‘**great deal of confidence**’, how much confidence do you have in the following institutions?

|  | No confidence at all | Great deal of confidence |
| --- | --- | --- |

|  | 0 | 1 | 2 | 3 | 4 | 5 | 6 | 7 | 8 | 9 | 10 |
| --- | --- | --- | --- | --- | --- | --- | --- | --- | --- | --- | --- |

| The government () | 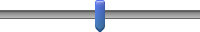 |
| --- | --- |
| Political parties () | 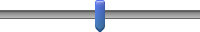 |
| Parliament () | 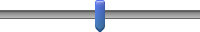 |
| The armed forces () | 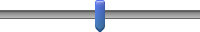 |
| The press () | 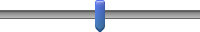 |
| Television () | 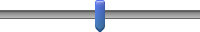 |
| Labour unions () | 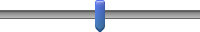 |
| The police () | 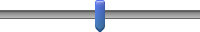 |
| The courts () | 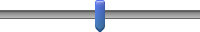 |
| The civil service () | 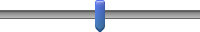 |
| Universities () | 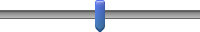 |

| Page Break |  |
| --- | --- |

| 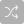 |
| --- |

populism How much do you agree or disagree with the following statements?

|  | Strongly agree (1) | Somewhat agree (2) | Neither agree nor disagree (3) | Somewhat disagree (4) | Strongly disagree (5) |
| --- | --- | --- | --- | --- | --- |
| The politicians in the House of Commons need to follow the will of the people. (1) |  |  |  |  |  |
| The people, and not politicians, should make our most important policy decisions. (2) |  |  |  |  |  |
| The political differences between the elite and the people are larger than the differences among the people. (3) |  |  |  |  |  |
| I would rather be represented by a citizen than by a specialized politician. (4) |  |  |  |  |  |
| Elected officials talk too much and take too little action. (5) |  |  |  |  |  |
| What people call “compromise” in politics is really just selling out on one’s principles. (6) |  |  |  |  |  |

End of Block: Scales

Start of Block: covid19

c19_preamble In this part of the survey, you will be asked about your experience with the coronavirus (COVID-19) pandemic.

| Page Break |  |
| --- | --- |

had_c19 Have you been infected with coronavirus (COVID-19) since the start of the pandemic?

- Yes (1)
- No (2)
- Unsure (3)

fam_c19 Besides you, has anyone in your household been infected with coronavirus (COVID-19) since the start of the pandemic?

- Yes (1)
- No (2)
- Unsure (3)

| Page Break |  |
| --- | --- |

vaccinated_c19 Have you received a coronavirus (COVID-19) vaccine? Please do not include any information about booster shots received. We will ask about booster shots later.

- Yes, I have received a one-shot vaccine. (1)
- Yes, I have received the first dose of a two-shot vaccine. (2)
- Yes, I have received two doses of a two-shot vaccine. (3)
- No, I have not received any vaccine doses. (4)

| Page Break |  |
| --- | --- |

Display this question:

If Have you received a coronavirus (COVID-19) vaccine? Please do not include any information about b... = No, I have not received any vaccine doses.

no_vaccine_c19 You selected ‘**No, I have not received any vaccine doses**’ in the previous step. Please select all reasons that apply to your answer.

- A coronavirus (COVID-19) vaccine was not available to me. (1)
- I was not eligible for a coronavirus (COVID-19) vaccine. (2)
- I did not want to receive a coronavirus (COVID-19) vaccine. (3)
- I did not have time to access a coronavirus (COVID-19) vaccine. (4)

| Page Break |  |
| --- | --- |

Display this question:

If Have you received a coronavirus (COVID-19) vaccine? Please do not include any information about b... != No, I have not received any vaccine doses.

booster_c19 Have you received a coronavirus (COVID-19) booster vaccine?

- Yes, I have received one or more shots of a coronavirus (COVID-19) booster vaccine. (1)
- No, I have not received a coronavirus (COVID-19) booster vaccine. (2)

| Page Break |  |
| --- | --- |

Display this question:

If Have you received a coronavirus (COVID-19) booster vaccine? = Yes, I have received one or more shots of a coronavirus (COVID-19) booster vaccine.

booster_second How many booster shots have you received?

▼ I have not received a booster shot. (1) ... More than 4 booster shots. (6)

| Page Break |  |
| --- | --- |

Display this question:

If Have you received a coronavirus (COVID-19) booster vaccine? = No, I have not received a coronavirus (COVID-19) booster vaccine.

no_booster_c19 You selected ‘**No, I have not received a coronavirus (COVID-19) booster vaccine**’ in the previous step. Please select all reasons that apply to your answer.

- A coronavirus (COVID-19) booster was not available to me. (1)
- I was not eligible for a coronavirus (COVID-19) booster. (2)
- I did not want to take a coronavirus (COVID-19) booster. (3)
- I did not have time to access a coronavirus (COVID-19) booster. (4)

| Page Break |  |
| --- | --- |

| 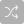 |
| --- |

actions_om_c19_1 On a scale of 0-10, where 0 means '**never**' and 10 means '**always**', how frequently do you currently engage in the following actions or behaviours in response to the coronavirus (COVID-19)?

|  | Never | Always | Not Applicable |
| --- | --- | --- | --- |

|  | 0 | 1 | 2 | 3 | 4 | 5 | 6 | 7 | 8 | 9 | 10 |
| --- | --- | --- | --- | --- | --- | --- | --- | --- | --- | --- | --- |

| Wear a face mask in indoor public spaces. () | 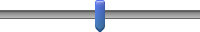 |
| --- | --- |
| Wear a face mask in outdoor public spaces. () | 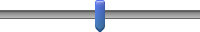 |
| Work, or attend school, from home. () | 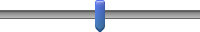 |
| Avoid going to pubs/bars/restaurants. () | 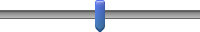 |
| Social distance in indoor public areas. () | 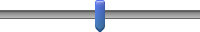 |

| Page Break |  |
| --- | --- |

| 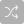 |
| --- |

actions_om_c19_2 On a scale of 0-10, where 0 means '**never**' and 10 means '**always'**, how frequently do you currently engage in the following actions or behaviours in response to the coronavirus (COVID-19)?

|  | Never | Always | Not Applicable |
| --- | --- | --- | --- |

|  | 0 | 1 | 2 | 3 | 4 | 5 | 6 | 7 | 8 | 9 | 10 |
| --- | --- | --- | --- | --- | --- | --- | --- | --- | --- | --- | --- |

| Avoid small indoor gatherings (less than 6 people). () | 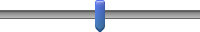 |
| --- | --- |
| Avoid large indoor gatherings (6 or more people). () | 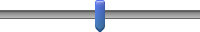 |
| Social distance in outdoor public areas. () | 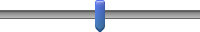 |
| Avoid small outdoor gatherings (less than 6 people). () | 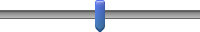 |
| Avoid large outdoor gatherings (6 or more people). () | 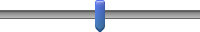 |

| Page Break |  |
| --- | --- |

vaccine_preamble Now you will be asked about your attitudes and beliefs towards vaccination and the coronavirus (COVID-19). There are no ‘right’ or ‘wrong’ answers. Please answer as honestly as you can.

| Page Break |  |
| --- | --- |

| 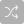 |
| --- |

trust_vaccines On a scale of 0-10, where **0** means ‘**strongly disagree**’ and **10** means ‘**strongly agree**’, how much do you agree or disagree, with the following statements?

|  | Strongly disagree | Strongly agree |
| --- | --- | --- |

|  | 0 | 1 | 2 | 3 | 4 | 5 | 6 | 7 | 8 | 9 | 10 |
| --- | --- | --- | --- | --- | --- | --- | --- | --- | --- | --- | --- |

| Vaccination is generally good for building immunity against the novel coronavirus that causes COVID-19. () | 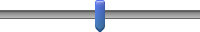 |
| --- | --- |
| A vaccine will protect those who receive it from possible health effects of coronavirus (COVID-19). () | 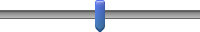 |
| Without a vaccine, I am likely to catch coronavirus (COVID-19). () | 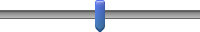 |
| I am worried about potential side effects of a coronavirus (COVID-19) vaccine. () | 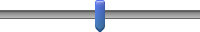 |
| Receiving a coronavirus (COVID-19) vaccine has improved my sense of belonging to my community. () | 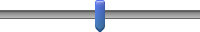 |
| Too much fuss is being made about the risk of coronavirus (COVID-19). () | 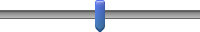 |

| Page Break |  |
| --- | --- |

| 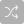 |
| --- |

ignore_community On a scale of 0-10, where **0** means ‘**strongly disagree**’ and **10** means ‘**strongly agree**’, how much do you agree or disagree with the following statement?

|  | Strongly disagree | Strongly agree |
| --- | --- | --- |

|  | 0 | 1 | 2 | 3 | 4 | 5 | 6 | 7 | 8 | 9 | 10 |
| --- | --- | --- | --- | --- | --- | --- | --- | --- | --- | --- | --- |

| Politicians usually ignore my community. () | 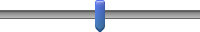 |
| --- | --- |

| Page Break |  |
| --- | --- |

| 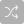 |
| --- |

lives_over_liberty On a scale of 0-10, where **0** means ‘**strongly disagree**’ and **10** means ‘**strongly agree**’, how much do you agree or disagree with the following statement?

|  | Strongly disagree | Strongly agree |
| --- | --- | --- |

|  | 0 | 1 | 2 | 3 | 4 | 5 | 6 | 7 | 8 | 9 | 10 |
| --- | --- | --- | --- | --- | --- | --- | --- | --- | --- | --- | --- |

| Saving lives is more important than personal liberty. () | 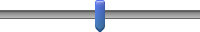 |
| --- | --- |

| Page Break |  |
| --- | --- |

trust_vaccines2 Generally speaking, would you say that coronavirus (COVID-19) vaccines can be trusted?

- Yes, coronavirus (COVID-19) vaccines can be trusted. (1)
- No, coronavirus (COVID-19) vaccines cannot be trusted. (2)

| Page Break |  |
| --- | --- |

trust_who Which of the following sources would you trust MOST to help you decide whether you would get a COVID-19 vaccine and/or the booster?

- Your doctor or healthcare provider (1)
- Your co-worker (2)
- Your employer (3)
- Prime Minister Boris Johnson (4)
- Your local public health authority (5)
- Your friends or family (6)
- Your neighbours (7)
- Your local community (8)
- Your pastor, priest, or other religious leader (9)
- Other (please specify) (10) __________________________________________________

End of Block: covid19

Start of Block: Media consumption

| 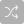 |
| --- |

covid_news_source What is your primary source for news on the coronavirus (COVID-19) pandemic?

- Newspapers (websites or in print) (1)
- Television news (2)
- Radio (3)
- Social media (4)
- WhatsApp (5)
- WeChat (6)

| Page Break |  |
| --- | --- |

covid_news_volume How often have you read, listened to, or watched news related to the coronavirus (COVID-19) pandemic over the past week?

- Several times a day (1)
- Daily (2)
- Almost every day (3)
- A few times (4)
- Once (5)
- Never (6)

End of Block: Media consumption

Start of Block: Screen2

screen2 People are very busy these days and many do not have time to follow what goes on in the government. We are testing whether people read questions. To show that you’ve read this much, answer both “Extremely interested” and “Very interested”:

- Extremely disinterested (1)
- Very disinterested (2)
- Somewhat disinterested (3)
- Neither disinterested nor interested (4)
- Somewhat interested (5)
- Very interested (6)
- Extremely interested (7)

End of Block: Screen2

Start of Block: conjoint

conjoint_preamble_t Timing

First Click (1)

Last Click (2)

Page Submit (3)

Click Count (4)

conjoint_preamble We are interested in knowing what you think the government should do about COVID-19 vaccines.

Recently boosters are being offered by public health authorities to citizens to increase protection against the novel coronavirus and COVID-19. 

Imagine that, later in the year, your government will introduce a new vaccine policy that promotes boosters to protect the public from getting COVID-19 from new variants of the coronavirus. 

To gauge your views, we will show you several pairs of hypothetical government policies about COVID-19 vaccines. For each pair of hypothetical vaccine policies, please think about which vaccine policy you prefer. There are no ‘right’ or ‘wrong’ answers. Please answer as honestly as you can. 

| Page Break |  |
| --- | --- |

conjoint1_t Timing

First Click (1)

Last Click (2)

Page Submit (3)

Click Count (4)

mobile1 **If you are using your mobile phone for this survey, please turn the phone sideways to see the whole table.**

conjoint1   **Vaccine Policy A** **Vaccine Policy B** How will booster appointments be scheduled?  ${e://Field/schedule} ${e://Field/schedule1} Will you get reminders to receive the booster? ${e://Field/reminders} ${e://Field/reminders1} Will employers be allowed to require that employees must receive the booster? ${e://Field/mandate} ${e://Field/mandate1} Will the government issue fines if you do not receive your booster when eligible? ${e://Field/fines} ${e://Field/fines1}

prefer Which is your preferred choice of future vaccine policy?

- Vaccine Policy A (1)
- Vaccine Policy B (2)

support On a scale of 0-10, where **0** means you ‘**definitely do not support**’ this vaccine policy, and **10** means you ‘**definitely support**' this vaccine policy, how would you rate each vaccine policy?

|  | Definitely do not support | Definitely support |
| --- | --- | --- |

|  | 0 | 1 | 2 | 3 | 4 | 5 | 6 | 7 | 8 | 9 | 10 |
| --- | --- | --- | --- | --- | --- | --- | --- | --- | --- | --- | --- |

| Vaccine Policy A () | 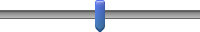 |
| --- | --- |
| Vaccine Policy B () | 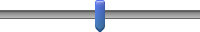 |

| Page Break |  |
| --- | --- |

conjoint2_t Timing

First Click (1)

Last Click (2)

Page Submit (3)

Click Count (4)

mobile2 **If you are using your mobile phone for this survey, please turn the phone sideways to see the whole table.**

conjoint2   **Vaccine Policy A** **Vaccine Policy B** How will booster appointments be scheduled?  ${e://Field/schedule2} ${e://Field/schedule3} Will you get reminders to receive the booster? ${e://Field/reminders2} ${e://Field/reminders3} Will employers be allowed to require that employees must receive the booster? ${e://Field/mandate2} ${e://Field/mandate3} Will the government issue fines if you do not receive your booster when eligible? ${e://Field/fines2} ${e://Field/fines3}

prefer2 Which is your preferred choice of future vaccine policy?

- Vaccine Policy A (1)
- Vaccine Policy B (2)

support2 On a scale of 0-10, where **0** means you ‘**definitely do not support**’ this vaccine policy, and **10** means you ‘**definitely support**' this vaccine policy, how would you rate each vaccine policy?

|  | Definitely do not support | Definitely support |
| --- | --- | --- |

|  | 0 | 1 | 2 | 3 | 4 | 5 | 6 | 7 | 8 | 9 | 10 |
| --- | --- | --- | --- | --- | --- | --- | --- | --- | --- | --- | --- |

| Vaccine Policy A () | 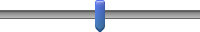 |
| --- | --- |
| Vaccine Policy B () | 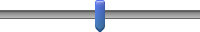 |

| Page Break |  |
| --- | --- |

conjoint3_t Timing

First Click (1)

Last Click (2)

Page Submit (3)

Click Count (4)

mobile3 **If you are using your mobile phone for this survey, please turn the phone sideways to see the whole table.**

conjoint3   **Vaccine Policy A** **Vaccine Policy B** How will booster appointments be scheduled?  ${e://Field/schedule4} ${e://Field/schedule5} Will you get reminders to receive the booster? ${e://Field/reminders4} ${e://Field/reminders5} Will employers be allowed to require that employees must receive the booster? ${e://Field/mandate4} ${e://Field/mandate5} Will the government issue fines if you do not receive your booster when eligible? ${e://Field/fines4} ${e://Field/fines5}

prefer3 Which is your preferred choice of future vaccine policy?

- Vaccine Policy A (1)
- Vaccine Policy B (2)

support3 On a scale of 0-10, where **0** means you ‘**definitely do not support**’ this vaccine policy, and **10** means you ‘**definitely support**' this vaccine policy, how would you rate each vaccine policy?

|  | Definitely do not support | Definitely support |
| --- | --- | --- |

|  | 0 | 1 | 2 | 3 | 4 | 5 | 6 | 7 | 8 | 9 | 10 |
| --- | --- | --- | --- | --- | --- | --- | --- | --- | --- | --- | --- |

| Vaccine Policy A () | 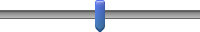 |
| --- | --- |
| Vaccine Policy B () | 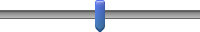 |

| Page Break |  |
| --- | --- |

tradeoff_t Timing

First Click (1)

Last Click (2)

Page Submit (3)

Click Count (4)

| 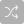 |
| --- |

tradeoff
Imagine the following scenario: In October of 2022, a new variant emerges which, like Omicron, is highly contagious. New vaccine boosters are developed. These boosters provide ${e://Field/effective} protection against infection from the new variant.

The government in your country would like individuals to take this booster shot. Which of the following policies would you support?

|  | Yes (3) | No (4) | Unsure (5) |
| --- | --- | --- | --- |
| No one should be forced to take the booster, but it should be available to anyone who wants it. (1) |  |  |  |
| Those who do not take the booster should be stopped from entering any indoor public spaces (e.g., restaurants, entertainment venues) and/or using public transport. (5) |  |  |  |
| Employers should require their employees to get the booster. (6) |  |  |  |
| Those who do not take the booster should be fined by the government. (7) |  |  |  |

End of Block: conjoint

Start of Block: screens3

screen3 Most modern theories of decision making recognize that decisions do not take place in a vacuum. Individual preferences and knowledge, along with situational variables can greatly impact the decision process. To demonstrate that you’ve read this much, just go ahead and select both red and green among the alternatives below, no matter what your favourite colour is. Yes, ignore the question below and select both of those options.

What is your favourite colour?

- White (1)
- Black (2)
- Red (3)
- Pink (4)
- Green (5)
- Blue (6)

End of Block: screens3

Start of Block: treatment1

timer1 Timing

First Click (1)

Last Click (2)

Page Submit (3)

Click Count (4)

preamble1 Imagine this: In October 2022, COVID-19 cases are rising in your area. The government is making another vaccine booster shot freely available to you as winter is approaching. 

In this scenario, **the government leaves it to every adult living in your country to choose whether they should get this vaccine booster shot or not**. If you want a booster, you will have to call your local clinic to schedule a booster appointment.

annual1 In this scenario, how likely is it that you would get this booster?

- Very likely (1)
- Somewhat likely (2)
- Slightly likely (3)
- Slightly unlikely (4)
- Somewhat unlikely (5)
- Very unlikely (6)

Display this question:

If How many children under the age of 18 are you the parent or guardian of? != 0

And How many children under the age of 18 are you the parent or guardian of? , 0 Is Displayed

child1 In this scenario, how likely is it that you would allow your child/children under the age of 18 to get this booster?

- Very likely (1)
- Somewhat likely (2)
- Slightly likely (3)
- Slightly unlikely (4)
- Somewhat unlikely (5)
- Very unlikely (6)

Display this question:

If Are you a parent or guardian to any children? = No

Or How many children under the age of 18 are you the parent or guardian of? = 0

child_all_1 In this scenario, if you had a child under the age of 18, how likely is it that you would allow your child to get this booster?

- Very likely (1)
- Somewhat likely (2)
- Slightly likely (3)
- Slightly unlikely (4)
- Somewhat unlikely (5)
- Very unlikely (6)

approve1 Do you approve or disapprove of the government’s action in this scenario? Please answer using the scale below, where **0** means ‘**I disapprove of the government’s action**’ and **10** means ‘**I approve of the government’s action**’.

|  | I disapprove of the government's action | I approve of the government's action |
| --- | --- | --- |

|  | 0 | 1 | 2 | 3 | 4 | 5 | 6 | 7 | 8 | 9 | 10 |
| --- | --- | --- | --- | --- | --- | --- | --- | --- | --- | --- | --- |

| () | 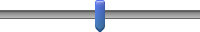 |
| --- | --- |

action1 In this scenario, do you think the government is doing too little, just the right amount, or too much to manage the coronavirus (COVID-19) pandemic in your country?

- Too little (1)
- Just the right amount (2)
- Too much (3)

| Page Break |  |
| --- | --- |

manicheck_1_t Timing

First Click (1)

Last Click (2)

Page Submit (3)

Click Count (4)

| 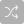 |
| --- |

manicheck_1 In this scenario, what did the government do to manage rising COVID-19 cases in your area?

- The government leaves it to every adult living in your country to choose whether they should get this vaccine booster shot or not. (1)
- The government announces that every adult living in your country will be automatically enrolled to receive this vaccine booster shot at a local clinic. (5)
- The government announces that every living adult in your country will be required to receive this vaccine booster to travel. (6)
- The government announces that it will fine adults living in your country who do not receive this vaccine booster. (7)

End of Block: treatment1

Start of Block: treatment2

timer2 Timing

First Click (1)

Last Click (2)

Page Submit (3)

Click Count (4)

preamble2 Imagine this: In October 2022, COVID-19 cases are rising in your area. The government is making another vaccine booster shot freely available to you as winter is approaching. 

In this scenario, **the government announces that every adult living in your country will be automatically enrolled to receive this vaccine booster shot at a local clinic**. Your local clinic will call you to schedule a booster appointment at a convenient date and time. You can opt out of this automatic enrolment if you wish.

annual2 In this scenario, how likely is it that you would get this booster?

- Very likely (1)
- Somewhat likely (2)
- Slightly likely (3)
- Slightly unlikely (4)
- Somewhat unlikely (5)
- Very unlikely (6)

Display this question:

If How many children under the age of 18 are you the parent or guardian of? != 0

And How many children under the age of 18 are you the parent or guardian of? , 0 Is Displayed

child2 In this scenario, how likely is it that you would allow your child/children under the age of 18 to get this booster?

- Very likely (1)
- Somewhat likely (2)
- Slightly likely (3)
- Slightly unlikely (4)
- Somewhat unlikely (5)
- Very unlikely (6)

Display this question:

If Are you a parent or guardian to any children? = No

Or How many children under the age of 18 are you the parent or guardian of? = 0

child_all_2 In this scenario, if you had a child under the age of 18, how likely is it that you would allow your child to get this booster?

- Very likely (1)
- Somewhat likely (2)
- Slightly likely (3)
- Slightly unlikely (4)
- Somewhat unlikely (5)
- Very unlikely (6)

approve2 Do you approve or disapprove of the government’s action in this scenario? Please answer using the scale below, where **0** means ‘**I disapprove of the government’s action**’ and **10** means ‘**I approve of the government’s action**’.

|  | I disapprove of the government's action | I approve of the government’s action |
| --- | --- | --- |

|  | 0 | 1 | 2 | 3 | 4 | 5 | 6 | 7 | 8 | 9 | 10 |
| --- | --- | --- | --- | --- | --- | --- | --- | --- | --- | --- | --- |

| () | 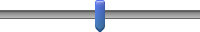 |
| --- | --- |

action2 In this scenario, do you think the government is doing too little, just the right amount, or too much to manage the coronavirus (COVID-19) pandemic in your country?

- Too little (1)
- Just the right amount (2)
- Too much (3)

| Page Break |  |
| --- | --- |

manicheck_2_t Timing

First Click (1)

Last Click (2)

Page Submit (3)

Click Count (4)

| 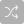 |
| --- |

manicheck_2 In this scenario, what did the government do to manage rising COVID-19 cases in your area?

- The government leaves it to every adult living in your country to choose whether they should get this vaccine booster shot or not. (1)
- The government announces that every adult living in your country will be automatically enrolled to receive this vaccine booster shot at a local clinic. (5)
- The government announces that every living adult in your country will be required to receive this vaccine booster to travel. (6)
- The government announces that it will fine adults living in your country who do not receive this vaccine booster. (7)

End of Block: treatment2

Start of Block: treatment3

timer3 Timing

First Click (1)

Last Click (2)

Page Submit (3)

Click Count (4)

preamble3 Imagine this: In October 2022, COVID-19 cases are rising in your area. The government is making another vaccine booster shot freely available to you as winter is approaching. 

In this scenario, **the government announces that** **every adult living in your country will be automatically enrolled to receive this vaccine booster shot at a local clinic**. Your local clinic will call you to schedule a booster appointment at a convenient date and time. You can opt out of this automatic enrolment if you wish.

| 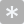 |
| --- |

text3 Please think about the government's actions in this scenario. Do you think this approach is appropriate? Do you think this approach will work for you? In at least one or two sentences, please write down your thoughts.

________________________________________________________________

________________________________________________________________

________________________________________________________________

________________________________________________________________

________________________________________________________________

annual3 In this scenario, how likely is it that you would get this booster?

- Very likely (1)
- Somewhat likely (2)
- Slightly likely (3)
- Slightly unlikely (4)
- Somewhat unlikely (5)
- Very unlikely (6)

Display this question:

If How many children under the age of 18 are you the parent or guardian of? != 0

And How many children under the age of 18 are you the parent or guardian of? , 0 Is Displayed

child3 In this scenario, how likely is it that you would allow your child/children under the age of 18 to get this booster?

- Very likely (1)
- Somewhat likely (2)
- Slightly likely (3)
- Slightly unlikely (4)
- Somewhat unlikely (5)
- Very unlikely (6)

Display this question:

If Are you a parent or guardian to any children? = No

Or How many children under the age of 18 are you the parent or guardian of? = 0

child_all_3 In this scenario, if you had a child under the age of 18, how likely is it that you would allow your child to get this booster?

- Very likely (1)
- Somewhat likely (2)
- Slightly likely (3)
- Slightly unlikely (4)
- Somewhat unlikely (5)
- Very unlikely (6)

approve3 Do you approve or disapprove of the government’s action in this scenario? Please answer using the scale below, where **0** means ‘**I disapprove of the government’s action**’ and **10** means ‘**I approve of the government’s action**’.

|  | I disapprove of the government’s action | I approve of the government’s action |
| --- | --- | --- |

|  | 0 | 1 | 2 | 3 | 4 | 5 | 6 | 7 | 8 | 9 | 10 |
| --- | --- | --- | --- | --- | --- | --- | --- | --- | --- | --- | --- |

| () | 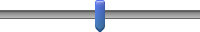 |
| --- | --- |

action3 In this scenario, do you think the government is doing too little, just the right amount, or too much to manage the coronavirus (COVID-19) pandemic in your country?

- Too little (1)
- Just the right amount (2)
- Too much (3)

| Page Break |  |
| --- | --- |

manicheck_3_t Timing

First Click (1)

Last Click (2)

Page Submit (3)

Click Count (4)

| 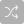 |
| --- |

manicheck_3 In this scenario, what did the government do to manage rising COVID-19 cases in your area?

- The government leaves it to every adult living in your country to choose whether they should get this vaccine booster shot or not. (1)
- The government announces that every adult living in your country will be automatically enrolled to receive this vaccine booster shot at a local clinic. (5)
- The government announces that every living adult in your country will be required to receive this vaccine booster to travel. (6)
- The government announces that it will fine adults living in your country who do not receive this vaccine booster. (7)

End of Block: treatment3

Start of Block: treatment4

timer4 Timing

First Click (1)

Last Click (2)

Page Submit (3)

Click Count (4)

preamble4 Imagine this: In October 2022, COVID-19 cases are rising in your area. The government is making another vaccine booster shot freely available to you as winter is approaching. 

In this scenario, **the government leaves it to every adult living in your country to choose whether they should get this vaccine booster shot or not**. If you want a booster, you will have to call your local clinic to schedule a booster appointment.

| 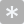 |
| --- |

text4 Please think about the government's actions in this scenario. Do you think this approach is appropriate? Do you think this approach will work for you? In at least one or two sentences, please write down your thoughts.

________________________________________________________________

________________________________________________________________

________________________________________________________________

________________________________________________________________

________________________________________________________________

annual4 In this scenario, how likely is it that you would get this booster?

- Very likely (1)
- Somewhat likely (2)
- Slightly likely (3)
- Slightly unlikely (4)
- Somewhat unlikely (5)
- Very unlikely (6)

Display this question:

If How many children under the age of 18 are you the parent or guardian of? != 0

And How many children under the age of 18 are you the parent or guardian of? , 0 Is Displayed

child4 In this scenario, how likely is it that you would allow your child/children under the age of 18 to get this booster?

- Very likely (1)
- Somewhat likely (2)
- Slightly likely (3)
- Slightly unlikely (4)
- Somewhat unlikely (5)
- Very unlikely (6)

Display this question:

If Are you a parent or guardian to any children? = No

Or How many children under the age of 18 are you the parent or guardian of? = 0

child_all_4 In this scenario, if you had a child under the age of 18, how likely is it that you would allow your child to get this booster?

- Very likely (1)
- Somewhat likely (2)
- Slightly likely (3)
- Slightly unlikely (4)
- Somewhat unlikely (5)
- Very unlikely (6)

approve4 Do you approve or disapprove of the government’s action in this scenario? Please answer using the scale below, where **0** means ‘**I disapprove of the government’s action**’ and **10** means ‘**I approve of the government’s action**’.

|  | I disapprove of the government’s action | I approve of the government’s action |
| --- | --- | --- |

|  | 0 | 1 | 2 | 3 | 4 | 5 | 6 | 7 | 8 | 9 | 10 |
| --- | --- | --- | --- | --- | --- | --- | --- | --- | --- | --- | --- |

| () | 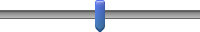 |
| --- | --- |

action4 In this scenario, do you think the government is doing too little, just the right amount, or too much to manage the coronavirus (COVID-19) pandemic in your country?

- Too little (1)
- Just the right amount (2)
- Too much (3)

| Page Break |  |
| --- | --- |

manicheck_4_t Timing

First Click (1)

Last Click (2)

Page Submit (3)

Click Count (4)

| 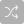 |
| --- |

manicheck_4 In this scenario, what did the government do to manage rising COVID-19 cases in your area?

- The government leaves it to every adult living in your country to choose whether they should get this vaccine booster shot or not. (1)
- The government announces that every adult living in your country will be automatically enrolled to receive this vaccine booster shot at a local clinic. (5)
- The government announces that every living adult in your country will be required to receive this vaccine booster to travel. (6)
- The government announces that it will fine adults living in your country who do not receive this vaccine booster. (7)

End of Block: treatment4

Start of Block: post-treatment

moregovernment_t Timing

First Click (1)

Last Click (2)

Page Submit (3)

Click Count (4)

moregovernment What else would you like the government to do to manage the coronavirus (COVID-19) pandemic in the scenario described above? 

In at least one or two sentences, please write down your thoughts in the text box below.

________________________________________________________________

________________________________________________________________

________________________________________________________________

________________________________________________________________

________________________________________________________________

| Page Break |  |
| --- | --- |

post_t Timing

First Click (1)

Last Click (2)

Page Submit (3)

Click Count (4)

post There is a great deal of uncertainty about how the future might look with the coronavirus still in circulation. Please describe what you expect to happen with the coronavirus pandemic in 2022 in the text box below.

________________________________________________________________

________________________________________________________________

________________________________________________________________

________________________________________________________________

________________________________________________________________

| Page Break |  |
| --- | --- |

future_scenario_1_t Timing

First Click (1)

Last Click (2)

Page Submit (3)

Click Count (4)

| 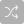 |
| --- |

future_scenario_1 Next, we'd like to know what you think will happen in the future with the coronavirus (COVID-19). On a scale of 0-10, where 0 means ‘completely unlikely to happen’ and 10 means ‘completely likely to happen’, please tell us how likely you think the following will happen **in the United Kingdom**. 

There are no ‘right’ or ‘wrong’ answers. Please answer as honestly as you can.

|  | Completely unlikely to happen | Completely likely to happen |
| --- | --- | --- |

|  | 0 | 1 | 2 | 3 | 4 | 5 | 6 | 7 | 8 | 9 | 10 |
| --- | --- | --- | --- | --- | --- | --- | --- | --- | --- | --- | --- |

| Compared to today, the number of COVID-19 related fatalities per day in the UK will be higher in 12 months. () | 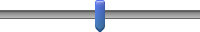 |
| --- | --- |
| Compared to today, the number of COVID-19 cases per day in the UK will be higher in 12 months. () | 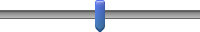 |
| COVID-19 vaccines available in the UK in 12 months will be more effective than the vaccines available today. () | 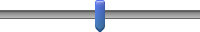 |
| People in the UK will be legally required to show proof of vaccination to access indoor public places in 12 months. () | 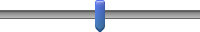 |
| Compared to today, public compliance with COVID-19 safety measures recommended by the UK government will be higher in 12 months. () | 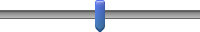 |
| In 12 months, the UK government will recommend at least one new booster shot. () | 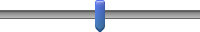 |

| Page Break |  |
| --- | --- |

future_scenario_2_t Timing

First Click (1)

Last Click (2)

Page Submit (3)

Click Count (4)

| 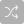 |
| --- |

future_scenario_2 Again, we'd like to know what you think will happen in the future with the coronavirus (COVID-19). On a scale of 0-10, where 0 means ‘completely unlikely to happen’ and 10 means ‘completely likely to happen’ please tell us how likely you think the following will happen **in the United Kingdom**. 

There are no ‘right’ or ‘wrong’ answers. Please answer as honestly as you can.

|  | Completely unlikely to happen | Completely likely to happen |
| --- | --- | --- |

|  | 0 | 1 | 2 | 3 | 4 | 5 | 6 | 7 | 8 | 9 | 10 |
| --- | --- | --- | --- | --- | --- | --- | --- | --- | --- | --- | --- |

| In 12 months, COVID-19 booster shots will be mandatory in the UK. () | 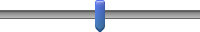 |
| --- | --- |
| There will be more restrictions on international travel to and from the UK in 12 months. () | 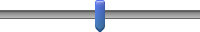 |
| Compared to today, people in the UK will socialise more in-person in 12 months. () | 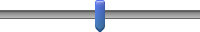 |
| People in the UK will be legally required to wear masks in indoor public places in 12 months. () | 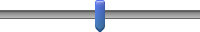 |
| Compared to today, people in the UK will work more often from home in 12 months. () | 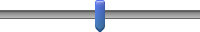 |

End of Block: post-treatment

UK-Wave2

Survey Flow

EmbeddedData

psidValue will be set from Panel or URL.

BlockRandomizer: 1 -

EmbeddedData

think = 1

EmbeddedData

think = 0

BlockRandomizer: 1 -

EmbeddedData

between_subjects = control

EmbeddedData

between_subjects = default_schedule

EmbeddedData

between_subjects = text_reminder

EmbeddedData

between_subjects = norm_nudge

BlockRandomizer: 1 -

EmbeddedData

booster_between = less_effective

EmbeddedData

booster_between = as_effective

EmbeddedData

booster_between = more_effective

Standard: ethics (2 Questions)

EmbeddedData

javascript_checkValue will be set from Panel or URL.

Branch: New Branch

If

If javascript_check Is Empty

EndSurvey: Advanced

Branch: New Branch

If

If Thank you for taking the time to participate in this study.    Please note that you need to be 18... I DO NOT give my consent to participate in this research study. Is Selected

EndSurvey: Advanced

Branch: New Branch

If

If What is your age? Please enter as a number (e.g., 25). Text Response Is Less Than or Equal to 17

EndSurvey: Advanced

Standard: captcha (1 Question)

Standard: Screens1 (1 Question)

Branch: New Branch

If

If Help us keep track of who is paying attention - please select “somewhat disagree” in the options... Somewhat disagree Is Not Selected

EndSurvey: Advanced

Standard: Demographics (14 Questions)

Branch: New Branch

If

If Do you currently live in the United Kingdom? No Is Selected

EndSurvey: Advanced

Standard: Party ID (3 Questions)

Standard: Scales (5 Questions)

Standard: covid19 (17 Questions)

Standard: Media consumption (2 Questions)

Standard: Screen2 (1 Question)

Standard: between_subjects (14 Questions)

Standard: screens3 (1 Question)

Standard: conjoint (14 Questions)

Standard: mechanisms (5 Questions)

Standard: booster_bs (11 Questions)

Standard: manip_check (1 Question)

Standard: post-treatment (6 Questions)

Standard: serious (3 Questions)

Standard: feedback (1 Question)

EmbeddedData

schedule1Value will be set from Panel or URL.

schedule2Value will be set from Panel or URL.

schedule3Value will be set from Panel or URL.

schedule4Value will be set from Panel or URL.

schedule5Value will be set from Panel or URL.

schedule6Value will be set from Panel or URL.

reminders1Value will be set from Panel or URL.

reminders2Value will be set from Panel or URL.

reminders3Value will be set from Panel or URL.

reminders4Value will be set from Panel or URL.

reminders5Value will be set from Panel or URL.

reminders6Value will be set from Panel or URL.

campaign1Value will be set from Panel or URL.

campaign2Value will be set from Panel or URL.

campaign3Value will be set from Panel or URL.

campaign4Value will be set from Panel or URL.

campaign5Value will be set from Panel or URL.

campaign6Value will be set from Panel or URL.

mandate1Value will be set from Panel or URL.

mandate2Value will be set from Panel or URL.

mandate3Value will be set from Panel or URL.

mandate4Value will be set from Panel or URL.

mandate5Value will be set from Panel or URL.

mandate6Value will be set from Panel or URL.

fines1Value will be set from Panel or URL.

fines2Value will be set from Panel or URL.

fines3Value will be set from Panel or URL.

fines4Value will be set from Panel or URL.

fines5Value will be set from Panel or URL.

fines6Value will be set from Panel or URL.

EmbeddedData

mechanismValue will be set from Panel or URL.

EmbeddedData

Q_TerminateFlagValue will be set from Panel or URL.

EndSurvey: Advanced

| Page Break |  |
| --- | --- |

Start of Block: ethics

| 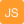 |
| --- |

ethics
 Thank you for taking the time to participate in this study.    Please note that you need to be 18+ and speak English fluently, otherwise please don't partake. The study should take around 15 minutes to complete. In the study, you will be asked a series of questions about vaccines. You will be paid for your participation in the survey. Just make sure to read all the instructions carefully and try your best.    NOTE: You can withdraw from the study at any stage without providing an explanation. Your privacy is very important, so we always use anonymised data. Results from this work may be written up for publication in a peer reviewed journal. However, individual data will never be published, and we will not hold personal identifiers. This project is in line with the ethical guidelines established by the Research Ethics Committee of King’s College London.   For more details about this research project, please see this information sheet.
 
If you have any questions you would like to ask before starting the survey, please feel free to contact Professor Peter John, King’s College London: peter.john@kcl.ac.uk. 
 
Please note in this survey we would like to ask some questions that may be perceived as sensitive, such as gender, ethnicity, political orientation, and religion. Providing information in response to these questions is entirely voluntary and you may withdraw your consent at any time. If you are happy to participate, please choose "I give my consent to participate in this research study."

- I DO NOT give my consent to participate in this research study. (1)
- I give my consent to participate in this research study. (2)

Skip To: End of Block If Thank you for taking the time to participate in this study.    Please note that you need to be 18... = I DO NOT give my consent to participate in this research study.

| Page Break |  |
| --- | --- |

| 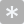 |
| --- |

yob What is your age? Please enter as a number (e.g., 25).

________________________________________________________________

Skip To: End of Block If Condition: What is your age? Please en... Is Less Than or Equal to 17. Skip To: End of Block.

End of Block: ethics

Start of Block: captcha

captcha Before you proceed to the survey, please complete the Captcha below.

End of Block: captcha

Start of Block: Screens1

screen1 Help us keep track of who is paying attention - please select “somewhat disagree” in the options below.

- Strongly disagree (1)
- Somewhat disagree (2)
- Neither agree nor disagree (3)
- Somewhat agree (4)
- Strongly agree (5)

Skip To: End of Block If Help us keep track of who is paying attention - please select “somewhat disagree” in the options... = Strongly disagree

Skip To: End of Block If Help us keep track of who is paying attention - please select “somewhat disagree” in the options... = Neither agree nor disagree

Skip To: End of Block If Help us keep track of who is paying attention - please select “somewhat disagree” in the options... = Somewhat agree

Skip To: End of Block If Help us keep track of who is paying attention - please select “somewhat disagree” in the options... = Strongly agree

End of Block: Screens1

Start of Block: Demographics

country Do you currently live in the United Kingdom?

- Yes (1)
- No (2)

Skip To: End of Block If Do you currently live in the United Kingdom? = No

| Page Break |  |
| --- | --- |

subnat_region Which region do you currently live in?

- East Anglia (1)
- East Midlands (3)
- London (4)
- North East (5)
- North West (6)
- Northern Ireland (7)
- Scotland (8)
- South East (9)
- South West (10)
- Wales (11)
- West Midlands (12)
- Yorkshire & Humberside (13)
- Prefer not to answer (2)

| Page Break |  |
| --- | --- |

gender Are you...

- A man (1)
- A woman (2)
- Non-binary (3)
- Another gender (please specify) (4) __________________________________________________
- Prefer not to answer (5)

| Page Break |  |
| --- | --- |

education What is the highest level of education you have achieved?

- Combined Junior and Infant School/ Infant School (1)
- Junior School (11)
- Comprehensive School (12)
- Comprehensive School (GCSE)/ Secondary Modern (GCSE)/ Grammar School (GCSE)/ City Technology College (GCSE)/ Sixth Form (13)
- College/ Institution of Higher education (14)
- Open College - College of Technology - Institute/ Teacher Training College (15)
- University/ Open University (16)
- Prefer not to answer (2)

| Page Break |  |
| --- | --- |

citizen Are you a citizen of the United Kingdom?

- Yes (1)
- No (2)

| Page Break |  |
| --- | --- |

parent_screen Are you a parent or guardian to any children?

- Yes (1)
- No (2)

| Page Break |  |
| --- | --- |

Display this question:

If Are you a parent or guardian to any children? = Yes

| 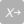 |
| --- |

children How many children under the age of 18 are you the parent or guardian of?

▼ 0 (4) ... 20 or more (24)

| Page Break |  |
| --- | --- |

| 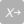 |
| --- |

children_u18 Are there any children under the age of 18 living in your household?

▼ 0 (1) ... 20 or more (21)

| Page Break |  |
| --- | --- |

pers_ethn Which of the following best describes your ethnicity?

- White: British (1)
- White: Irish (2)
- White: Other (3)
- Mixed: White and Black Caribbean (4)
- Mixed: White and Black African (5)
- Mixed: White and Asian (6)
- Mixed: Other mixed background (7)
- Black or Black British: African (8)
- Black or Black British: Caribbean (9)
- Black or Black British: Any other Black background (10)
- Asian or Asian British: Indian (11)
- Asian or Asian British: Pakistani (12)
- Asian or Asian British: Bangladeshi (13)
- Asian or Asian British: Other Asian background (14)
- Chinese (15)
- Other ethnic group not represented by these options (please specify) (16) __________________________________________________
- Do not wish to say (17)

| Page Break |  |
| --- | --- |

| 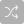 | 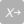 |
| --- | --- |

ethnicity Which of the following places best describes where your parents are from? Please select all that apply.

- The UK (1)
- Europe (outside of the UK) (2)
- Africa (3)
- Asia (4)
- Middle East (5)
- Oceania (6)
- North America (7)
- Central America (8)
- South America (9)
- The Caribbean (10)
- Other (11)
- ⊗Don’t Know (12)
- ⊗Prefer not to say (13)

| Page Break |  |
| --- | --- |

employment What is your employment status? Please select as many as applicable.

- Working for pay full-time (1)
- Working for pay part-time (2)
- Self-employed (3)
- Retired (4)
- Unemployed / Looking for work (5)
- Student (6)
- Caring for family (7)
- Other (Please specify) (8) __________________________________________________

| Page Break |  |
| --- | --- |

income What was your total household income, before taxes, for the year 2021?

- No income (1)
- £1 - £4,400 (2)
- £4,401 - £8,800 (4)
- £8,801 - £17,600 (5)
- £17,601 - £26,400 (6)
- £26,401 - £35,200 (7)
- £35,201 - £52,800 (8)
- £52,801 - £64,500 (9)
- £64,501 - £88,000 (10)
- £88,001 - £117,300 (11)
- More than £117,300 (12)
- Don't know / prefer not to answer (3)

| Page Break |  |
| --- | --- |

urban_rural Which of the following best describes the place where you now live…

- A large city (1)
- A suburb near a large city (2)
- A small city (3)
- A town (4)
- A rural area (5)

| Page Break |  |
| --- | --- |

religiosity In your life, you would say religion is:

- Very important (1)
- Somewhat important (2)
- Not very important (3)
- Not at all important (4)

End of Block: Demographics

Start of Block: Party ID

| 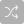 | 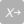 |
| --- | --- |

partyid In politics, do you usually think of yourself as a(n):

- Conservative Party (1)
- Labour Party (2)
- Scottish National Party (3)
- Liberal Democrats (4)
- Democratic Unionist Party (5)
- Sinn Féin (6)
- Plaid Cymru (7)
- Social Democratic and Labour Party (8)
- Green Party (9)
- Alliance Party of Northern Ireland (10)
- Another party (specify): (11) __________________________________________________
- No party (12)
- Don't know (13)

| Page Break |  |
| --- | --- |

Display this question:

If In politics, do you usually think of yourself as a(n): = Conservative Party

Or In politics, do you usually think of yourself as a(n): = Labour Party

Or In politics, do you usually think of yourself as a(n): = Scottish National Party

Or In politics, do you usually think of yourself as a(n): = Liberal Democrats

Or In politics, do you usually think of yourself as a(n): = Democratic Unionist Party

Or In politics, do you usually think of yourself as a(n): = Sinn Féin

Or In politics, do you usually think of yourself as a(n): = Plaid Cymru

Or In politics, do you usually think of yourself as a(n): = Social Democratic and Labour Party

Or In politics, do you usually think of yourself as a(n): = Green Party

Or In politics, do you usually think of yourself as a(n): = Alliance Party of Northern Ireland

partyid_strength How strongly ${partyid/ChoiceGroup/SelectedChoices} do you feel?

- Very strongly (1)
- Fairly strongly (2)
- Not very strongly (3)
- Don't know (4)

| Page Break |  |
| --- | --- |

Display this question:

If In politics, do you usually think of yourself as a(n): = Another party (specify):

And And In politics, do you usually think of yourself as a(n): Text Response Is Not Empty

partyid_strength How strongly ${partyid/ChoiceTextEntryValue/2} do you feel?

- Very strongly (1)
- Fairly strongly (2)
- Not very strongly (3)
- Don't know (4)

End of Block: Party ID

Start of Block: Scales

risk On a scale of 0-10, where **0** means you are ‘**completely unwilling to take risks’** and **10** means you are ‘**very willing to take risks’** in general, how willing or unwilling are you to take risks?

|  | Completely unwilling to take risks | Very willing to take risks |
| --- | --- | --- |

|  | 0 | 1 | 2 | 3 | 4 | 5 | 6 | 7 | 8 | 9 | 10 |
| --- | --- | --- | --- | --- | --- | --- | --- | --- | --- | --- | --- |

| 1 () | 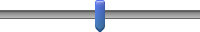 |
| --- | --- |

| Page Break |  |
| --- | --- |

lr_scale In political matters, people talk of the '**left**' and the '**right**'. How would you place your views on this scale, generally speaking?

|  | Left | Right |
| --- | --- | --- |

|  | 0 | 1 | 2 | 3 | 4 | 5 | 6 | 7 | 8 | 9 | 10 |
| --- | --- | --- | --- | --- | --- | --- | --- | --- | --- | --- | --- |

| 1 () | 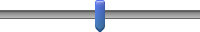 |
| --- | --- |

| Page Break |  |
| --- | --- |

pol_system_t Timing

First Click (1)

Last Click (2)

Page Submit (3)

Click Count (4)

| 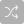 |
| --- |

pol_system On a scale of 0-10, where **0** means you have ‘**no confidence at all**’ and a **10** means you have a ‘**great deal of confidence**’, how much confidence do you have in the following institutions?

|  | No confidence at all | Great deal of confidence |
| --- | --- | --- |

|  | 0 | 1 | 2 | 3 | 4 | 5 | 6 | 7 | 8 | 9 | 10 |
| --- | --- | --- | --- | --- | --- | --- | --- | --- | --- | --- | --- |

| The government () | 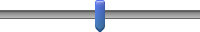 |
| --- | --- |
| Political parties () | 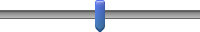 |
| Parliament () | 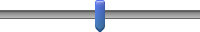 |
| The armed forces () | 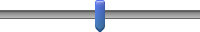 |
| The press () | 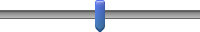 |
| Television () | 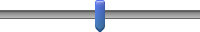 |
| Labour unions () | 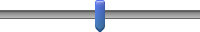 |
| The police () | 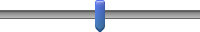 |
| The courts () | 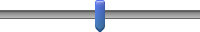 |
| The civil service () | 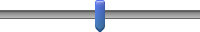 |
| Universities () | 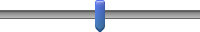 |

| Page Break |  |
| --- | --- |

| 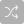 |
| --- |

populism How much do you agree or disagree with the following statements?

|  | Strongly agree (1) | Somewhat agree (2) | Neither agree nor disagree (3) | Somewhat disagree (4) | Strongly disagree (5) |
| --- | --- | --- | --- | --- | --- |
| The politicians in the House of Commons need to follow the will of the people. (1) |  |  |  |  |  |
| The people, and not politicians, should make our most important policy decisions. (2) |  |  |  |  |  |
| The political differences between the elite and the people are larger than the differences among the people. (3) |  |  |  |  |  |
| I would rather be represented by a citizen than by a specialized politician. (4) |  |  |  |  |  |
| Elected officials talk too much and take too little action. (5) |  |  |  |  |  |
| What people call “compromise” in politics is really just selling out on one’s principles. (6) |  |  |  |  |  |

End of Block: Scales

Start of Block: covid19

c19_preamble In this part of the survey, you will be asked about your experience with the coronavirus (COVID-19) pandemic.

| Page Break |  |
| --- | --- |

had_c19 Have you been infected with coronavirus (COVID-19) since the start of the pandemic?

- Yes (1)
- No (2)
- Unsure (3)

fam_c19 Besides you, has anyone in your household been infected with coronavirus (COVID-19) since the start of the pandemic?

- Yes (1)
- No (2)
- Unsure (3)

| Page Break |  |
| --- | --- |

vaccinated_c19 Have you received a coronavirus (COVID-19) vaccine? Please do not include any information about booster shots received. We will ask about booster shots later.

- Yes, I have received a one-shot vaccine. (1)
- Yes, I have received the first dose of a two-shot vaccine. (2)
- Yes, I have received two doses of a two-shot vaccine. (3)
- No, I have not received any vaccine doses. (4)

| Page Break |  |
| --- | --- |

Display this question:

If Have you received a coronavirus (COVID-19) vaccine? Please do not include any information about b... = No, I have not received any vaccine doses.

no_vaccine_c19 You selected ‘**No, I have not received any vaccine doses**’ in the previous step. Please select all reasons that apply to your answer.

- A coronavirus (COVID-19) vaccine was not available to me. (1)
- I was not eligible for a coronavirus (COVID-19) vaccine. (2)
- I did not want to receive a coronavirus (COVID-19) vaccine. (3)
- I did not have time to access a coronavirus (COVID-19) vaccine. (4)

| Page Break |  |
| --- | --- |

Display this question:

If Have you received a coronavirus (COVID-19) vaccine? Please do not include any information about b... != No, I have not received any vaccine doses.

booster_c19 Have you received a coronavirus (COVID-19) booster vaccine?

- Yes, I have received one or more shots of a coronavirus (COVID-19) booster vaccine. (1)
- No, I have not received a coronavirus (COVID-19) booster vaccine. (2)

| Page Break |  |
| --- | --- |

Display this question:

If Have you received a coronavirus (COVID-19) booster vaccine? = Yes, I have received one or more shots of a coronavirus (COVID-19) booster vaccine.

booster_second How many booster shots have you received?

▼ I have not received a booster shot. (1) ... More than 4 booster shots. (6)

| Page Break |  |
| --- | --- |

Display this question:

If Have you received a coronavirus (COVID-19) booster vaccine? = No, I have not received a coronavirus (COVID-19) booster vaccine.

no_booster_c19 You selected ‘**No, I have not received a coronavirus (COVID-19) booster vaccine**’ in the previous step. Please select all reasons that apply to your answer.

- A coronavirus (COVID-19) booster was not available to me. (1)
- I was not eligible for a coronavirus (COVID-19) booster. (2)
- I did not want to take a coronavirus (COVID-19) booster. (3)
- I did not have time to access a coronavirus (COVID-19) booster. (4)

| Page Break |  |
| --- | --- |

| 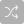 |
| --- |

actions_om_c19_1 On a scale of 0-10, where 0 means '**never**' and 10 means '**always**', how frequently do you currently engage in the following actions or behaviours in response to the coronavirus (COVID-19)?

|  | Never | Always | Not Applicable |
| --- | --- | --- | --- |

|  | 0 | 1 | 2 | 3 | 4 | 5 | 6 | 7 | 8 | 9 | 10 |
| --- | --- | --- | --- | --- | --- | --- | --- | --- | --- | --- | --- |

| Wear a face mask in indoor public spaces. () | 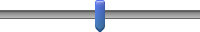 |
| --- | --- |
| Wear a face mask in outdoor public spaces. () | 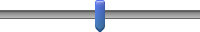 |
| Work, or attend school, from home. () |  |
| Avoid going to pubs/bars/restaurants. () |  |
| Social distance in indoor public areas. () |  |

| Page Break |  |
| --- | --- |

|  |
| --- |

actions_om_c19_2 On a scale of 0-10, where 0 means '**never**' and 10 means '**always'**, how frequently do you currently engage in the following actions or behaviours in response to the coronavirus (COVID-19)?

|  | Never | Always | Not Applicable |
| --- | --- | --- | --- |

|  | 0 | 1 | 2 | 3 | 4 | 5 | 6 | 7 | 8 | 9 | 10 |
| --- | --- | --- | --- | --- | --- | --- | --- | --- | --- | --- | --- |

| Avoid small indoor gatherings (less than 6 people). () |  |
| --- | --- |
| Avoid large indoor gatherings (6 or more people). () |  |
| Social distance in outdoor public areas. () |  |
| Avoid small outdoor gatherings (less than 6 people). () |  |
| Avoid large outdoor gatherings (6 or more people). () |  |

| Page Break |  |
| --- | --- |

vaccine_preamble Now you will be asked about your attitudes and beliefs towards vaccination and the coronavirus (COVID-19). There are no ‘right’ or ‘wrong’ answers. Please answer as honestly as you can.

| Page Break |  |
| --- | --- |

|  |
| --- |

trust_vaccines On a scale of 0-10, where **0** means ‘**strongly disagree**’ and **10** means ‘**strongly agree**’, how much do you agree or disagree, with the following statements?

|  | Strongly disagree | Strongly agree |
| --- | --- | --- |

|  | 0 | 1 | 2 | 3 | 4 | 5 | 6 | 7 | 8 | 9 | 10 |
| --- | --- | --- | --- | --- | --- | --- | --- | --- | --- | --- | --- |

| Vaccination is generally good for building immunity against the novel coronavirus that causes COVID-19. () |  |
| --- | --- |
| A vaccine will protect those who receive it from possible health effects of coronavirus (COVID-19). () |  |
| Without a vaccine, I am likely to catch coronavirus (COVID-19). () |  |
| I am worried about potential side effects of a coronavirus (COVID-19) vaccine. () |  |
| Receiving a coronavirus (COVID-19) vaccine has improved my sense of belonging to my community. () |  |
| Too much fuss is being made about the risk of coronavirus (COVID-19). () |  |

| Page Break |  |
| --- | --- |

|  |
| --- |

prevent_infection To the best of your knowledge, how effective are currently available vaccines at preventing COVID-19 infection?

- Very effective (1)
- Somewhat effective (2)
- Not very effective (3)
- Not at all effective (4)

| Page Break |  |
| --- | --- |

|  |
| --- |

ignore_community On a scale of 0-10, where **0** means ‘**strongly disagree**’ and **10** means ‘**strongly agree**’, how much do you agree or disagree with the following statement?

|  | Strongly disagree | Strongly agree |
| --- | --- | --- |

|  | 0 | 1 | 2 | 3 | 4 | 5 | 6 | 7 | 8 | 9 | 10 |
| --- | --- | --- | --- | --- | --- | --- | --- | --- | --- | --- | --- |

| Politicians usually ignore my community. () |  |
| --- | --- |

| Page Break |  |
| --- | --- |

|  |
| --- |

lives_over_liberty On a scale of 0-10, where **0** means ‘**strongly disagree**’ and **10** means ‘**strongly agree**’, how much do you agree or disagree with the following statement?

|  | Strongly disagree | Strongly agree |
| --- | --- | --- |

|  | 0 | 1 | 2 | 3 | 4 | 5 | 6 | 7 | 8 | 9 | 10 |
| --- | --- | --- | --- | --- | --- | --- | --- | --- | --- | --- | --- |

| Saving lives is more important than personal liberty. () |  |
| --- | --- |

| Page Break |  |
| --- | --- |

trust_vaccines2 Generally speaking, would you say that coronavirus (COVID-19) vaccines can be trusted?

- Yes, coronavirus (COVID-19) vaccines can be trusted. (1)
- No, coronavirus (COVID-19) vaccines cannot be trusted. (2)

| Page Break |  |
| --- | --- |

trust_who Which of the following sources would you trust MOST to help you decide whether you would get a COVID-19 vaccine and/or the booster?

- Your doctor or healthcare provider (1)
- Your co-worker (2)
- Your employer (3)
- Prime Minister Boris Johnson (4)
- Your local public health authority (5)
- Your friends or family (6)
- Your neighbours (7)
- Your local community (8)
- Your pastor, priest, or other religious leader (9)
- Other (please specify) (10) __________________________________________________

End of Block: covid19

Start of Block: Media consumption

|  |
| --- |

covid_news_source What is your primary source for news on the coronavirus (COVID-19) pandemic?

- Newspapers (websites or in print) (1)
- Television news (2)
- Radio (3)
- Social media (4)
- WhatsApp (5)
- WeChat (6)

| Page Break |  |
| --- | --- |

covid_news_volume How often have you read, listened to, or watched news related to the coronavirus (COVID-19) pandemic over the past week?

- Several times a day (1)
- Daily (2)
- Almost every day (3)
- A few times (4)
- Once (5)
- Never (6)

End of Block: Media consumption

Start of Block: Screen2

screen2 People are very busy these days and many do not have time to follow what goes on in the government. We are testing whether people read questions. To show that you’ve read this much, answer both “Extremely interested” and “Very interested”:

- Extremely disinterested (1)
- Very disinterested (2)
- Somewhat disinterested (3)
- Neither disinterested nor interested (4)
- Somewhat interested (5)
- Very interested (6)
- Extremely interested (7)

End of Block: Screen2

Start of Block: between_subjects

between_timer Timing

First Click (1)

Last Click (2)

Page Submit (3)

Click Count (4)

Display this question:

If between_subjects = control

control Imagine the following hypothetical scenario:
 In October 2022, COVID-19 cases are rising in your area. The government is making a new COVID-19 booster freely available. This new booster is safe, effective, and recommended for everyone – regardless of whether they got any previous doses of a COVID-19 vaccine.
  If you want to get this new booster, you can call your local health clinic to schedule an appointment at a convenient time.

Display this question:

If between_subjects = default_schedule

default_schedule Imagine the following hypothetical scenario: 
 In October 2022, COVID-19 cases are rising in your area. The government is making a new COVID-19 booster freely available. This new booster is safe, effective, and recommended for everyone – regardless of whether they got any previous doses of a COVID-19 vaccine.
 The government announces that local health clinics will call every adult to schedule an appointment for them to get this new booster. People can opt out of getting this new booster if they wish.
 If you want to get this new booster, you can select a convenient appointment time when your local health clinic calls you.

Display this question:

If between_subjects = text_reminder

text_reminder Imagine the following hypothetical scenario:  In October 2022, COVID-19 cases are rising in your area. The government is making a new COVID-19 booster freely available. This new booster is safe, effective, and recommended for everyone – regardless of whether they got any previous doses of a COVID-19 vaccine. 
 The government announces that local health clinics will automatically send every adult a text message to remind them that their dose of this new booster is ready.   
 If you want to get this new booster, you can call your local health clinic to schedule an appointment at a convenient time.

Display this question:

If between_subjects = norm_nudge

norm_nudge Imagine the following hypothetical scenario: 
 In October 2022, COVID-19 cases are rising in your area. The government is making a new COVID-19 booster freely available. This new booster is safe, effective, and recommended for everyone – regardless of whether they got any previous doses of a COVID-19 vaccine.
 The government announces that it estimates that 80% of people will choose to get this new booster.
 If you want to get this new booster, you can call your local health clinic to schedule an appointment at a convenient time.

Display this question:

If think = 1

|  |
| --- |

think Please think about whether you would like to get this new booster in this way based on what you just read. In at least one or two sentences, please write down your thoughts in the space provided below.

________________________________________________________________

________________________________________________________________

________________________________________________________________

________________________________________________________________

________________________________________________________________

annual In this scenario, how likely is it that you would get this booster?

- Very likely (1)
- Somewhat likely (2)
- Slightly likely (3)
- Slightly unlikely (4)
- Somewhat unlikely (5)
- Very unlikely (6)

Display this question:

If How many children under the age of 18 are you the parent or guardian of? != 0

And How many children under the age of 18 are you the parent or guardian of? , 0 Is Displayed

child In this scenario, how likely is it that you would allow your child/children under the age of 18 to get this booster?

- Very likely (1)
- Somewhat likely (2)
- Slightly likely (3)
- Slightly unlikely (4)
- Somewhat unlikely (5)
- Very unlikely (6)

Display this question:

If Are you a parent or guardian to any children? = No

Or How many children under the age of 18 are you the parent or guardian of? = 0

child_all In this scenario, if you had a child under the age of 18, how likely is it that you would allow your child to get this booster?

- Very likely (1)
- Somewhat likely (2)
- Slightly likely (3)
- Slightly unlikely (4)
- Somewhat unlikely (5)
- Very unlikely (6)

approve Do you approve or disapprove of the government’s action in this scenario? Please answer using the scale below, where **0** means ‘**I disapprove of the government’s action**’ and **10** means ‘**I approve of the government’s action**’.

|  | I disapprove of the government’s action | I approve of the government’s action |
| --- | --- | --- |

|  | 0 | 1 | 2 | 3 | 4 | 5 | 6 | 7 | 8 | 9 | 10 |
| --- | --- | --- | --- | --- | --- | --- | --- | --- | --- | --- | --- |

| () |  |
| --- | --- |

action In this scenario, do you think the government is doing too little, just the right amount, or too much to manage the coronavirus (COVID-19) pandemic in your country?

- Too little (1)
- Just the right amount (2)
- Too much (3)

| Page Break |  |
| --- | --- |

|  |  |
| --- | --- |

manip_check_bs In the hypothetical scenario you just read, which of the following – if any – did the government announce?

- The government announced that local health clinics will call every adult to schedule an appointment for them to get this new booster. (1)
- The government announced that local health clinics will automatically send every adult a text message to remind them that their dose of this new booster is ready. (2)
- The government announced that it estimates that 80% of people will choose to get this new booster. (3)
- None of the above. (4)

| Page Break |  |
| --- | --- |

moregovernment_t Timing

First Click (1)

Last Click (2)

Page Submit (3)

Click Count (4)

moregovernment What else would you like the government to do to manage the coronavirus (COVID-19) pandemic in the scenario described above? 

In at least one or two sentences, please write down your thoughts in the text box below.

________________________________________________________________

________________________________________________________________

________________________________________________________________

________________________________________________________________

________________________________________________________________

End of Block: between_subjects

Start of Block: screens3

screen3 Most modern theories of decision making recognize that decisions do not take place in a vacuum. Individual preferences and knowledge, along with situational variables can greatly impact the decision process. To demonstrate that you’ve read this much, just go ahead and select both red and green among the alternatives below, no matter what your favourite colour is. Yes, ignore the question below and select both of those options.

What is your favourite colour?

- White (1)
- Black (2)
- Red (3)
- Pink (4)
- Green (5)
- Blue (6)

End of Block: screens3

Start of Block: conjoint

conjoint_preamble Timing

First Click (1)

Last Click (2)

Page Submit (3)

Click Count (4)

|  |
| --- |

conjoint_preamble Next, we’d like you to imagine a new hypothetical scenario:
Several months from now, COVID-19 cases are increasing where you live. A new booster is being made freely available. It is safe, effective, and recommended for everyone – even if they already got previous COVID-19 vaccines. The government is considering different plans to encourage everyone to get this new booster. We are interested in knowing what you think the government should do. To help gauge your views, we will show you several pairs of hypothetical government plans and ask you what you think about them.

| Page Break |  |
| --- | --- |

conjoint1_t Timing

First Click (1)

Last Click (2)

Page Submit (3)

Click Count (4)

conjoint1 Please read the descriptions of the hypothetical government plans carefully. Each plan includes different combinations of policies as set out in the table below. After you’ve read about each plan, please indicate which of the two plans you prefer. (If you are using your mobile phone, please turn the phone sideways to see the whole table.)   **List of possible policies** **Vaccine Plan A** **Vaccine Plan B** Local health clinics will call every adult to offer to schedule an appointment for them to get this new booster.  ${e://Field/schedule1} ${e://Field/schedule2} Local health clinics will automatically send every adult a text message to remind them that their dose of this new booster is ready. ${e://Field/reminders1} ${e://Field/reminders2} The government will launch an advertising campaign that emphasizes how most people will likely choose to get this new booster. ${e://Field/campaign1} ${e://Field/campaign2} Employers will be allowed to require their employees to get this new booster. ${e://Field/mandate1} ${e://Field/mandate2} Eligible adults will be fined if they do not get this new booster. ${e://Field/fines1} ${e://Field/fines2}

prefer1 Which is your preferred choice of future vaccine plan?

- Vaccine Plan A (1)
- Vaccine Plan B (2)

support1 On a scale of 0-10, where **0** means you ‘**definitely do not support**’ this vaccine plan, and **10** means you ‘**definitely support**' this vaccine plan, how would you rate each vaccine plan?

|  | Definitely do not support | Definitely support |
| --- | --- | --- |

|  | 0 | 1 | 2 | 3 | 4 | 5 | 6 | 7 | 8 | 9 | 10 |
| --- | --- | --- | --- | --- | --- | --- | --- | --- | --- | --- | --- |

| Vaccine Plan A () |  |
| --- | --- |
| Vaccine Plan B () |  |

| Page Break |  |
| --- | --- |

conjoint2_t Timing

First Click (1)

Last Click (2)

Page Submit (3)

Click Count (4)

conjoint2 Please read the descriptions of the hypothetical government plans carefully. Each plan includes different combinations of policies as set out in the table below. After you’ve read about each plan, please indicate which of the two plans you prefer. (If you are using your mobile phone, please turn the phone sideways to see the whole table.)   **List of possible policies** **Vaccine Plan A** **Vaccine Plan B** Local health clinics will call every adult to offer to schedule an appointment for them to get this new booster.  ${e://Field/schedule3} ${e://Field/schedule4} Local health clinics will automatically send every adult a text message to remind them that their dose of this new booster is ready. ${e://Field/reminders3} ${e://Field/reminders4} The government will launch an advertising campaign that emphasizes how most people will likely choose to get this new booster. ${e://Field/campaign3} ${e://Field/campaign4} Employers will be allowed to require their employees to get this new booster. ${e://Field/mandate3} ${e://Field/mandate4} Eligible adults will be fined if they do not get this new booster.  ${e://Field/fines3} ${e://Field/fines4}

prefer2 Which is your preferred choice of future vaccine plan?

- Vaccine Plan A (1)
- Vaccine Plan B (2)

support2 On a scale of 0-10, where **0** means you ‘**definitely do not support**’ this vaccine plan, and **10** means you ‘**definitely support**' this vaccine plan, how would you rate each vaccine plan?

|  | Definitely do not support | Definitely support |
| --- | --- | --- |

|  | 0 | 1 | 2 | 3 | 4 | 5 | 6 | 7 | 8 | 9 | 10 |
| --- | --- | --- | --- | --- | --- | --- | --- | --- | --- | --- | --- |

| Vaccine Plan A () |  |
| --- | --- |
| Vaccine Plan B () |  |

| Page Break |  |
| --- | --- |

conjoint3_t Timing

First Click (1)

Last Click (2)

Page Submit (3)

Click Count (4)

conjoint3 Please read the descriptions of the hypothetical government plans carefully. Each plan includes different combinations of policies as set out in the table below. After you’ve read about each plan, please indicate which of the two plans you prefer. (If you are using your mobile phone, please turn the phone sideways to see the whole table.)   **List of possible policies** **Vaccine Plan A** **Vaccine Plan B** Local health clinics will call every adult to offer to schedule an appointment for them to get this new booster.  ${e://Field/schedule5} ${e://Field/schedule6} Local health clinics will automatically send every adult a text message to remind them that their dose of this new booster is ready. ${e://Field/reminders5} ${e://Field/reminders6} The government will launch an advertising campaign that emphasizes how most people will likely choose to get this new booster. ${e://Field/campaign5} ${e://Field/campaign6} Employers will be allowed to require their employees to get this new booster. ${e://Field/mandate5} ${e://Field/mandate6} Eligible adults will be fined if they do not get this new booster.  ${e://Field/fines5} ${e://Field/fines6}

prefer3 Which is your preferred choice of future vaccine plan?

- Vaccine Plan A (1)
- Vaccine Plan B (2)

support3 On a scale of 0-10, where **0** means you ‘**definitely do not support**’ this vaccine plan, and **10** means you ‘**definitely support**' this vaccine plan, how would you rate each vaccine plan?

|  | Definitely do not support | Definitely support |
| --- | --- | --- |

|  | 0 | 1 | 2 | 3 | 4 | 5 | 6 | 7 | 8 | 9 | 10 |
| --- | --- | --- | --- | --- | --- | --- | --- | --- | --- | --- | --- |

| Vaccine Plan A () |  |
| --- | --- |
| Vaccine Plan B () |  |

End of Block: conjoint

Start of Block: mechanisms

mech_timer Timing

First Click (1)

Last Click (2)

Page Submit (3)

Click Count (4)

mechanisms_prompt Next, we’d like to know more about what you think of the following policy in particular: 
 ${e://Field/mechanism}.

|  |
| --- |

mechanisms1
How effective do you think this policy would be at encouraging people to get this new booster?

- Very effective (1)
- Somewhat effective (2)
- Not very effective (3)
- Not at all effective (4)

|  |  |
| --- | --- |

mechanisms2 Which of the following statements comes closest to your view?

- This policy does too little to protect people’s freedom to decide for themselves whether to get this new booster. (1)
- This policy strikes an appropriate balance between protecting people’s freedom to decide for themselves whether to get this new booster and encouraging people to get this new booster. (2)
- This policy does too little to encourage people to get this new booster. (3)

mechanisms3 Do you have any further thoughts about this policy? If yes, please feel free to share your thoughts in the space provided below.

________________________________________________________________

________________________________________________________________

________________________________________________________________

________________________________________________________________

________________________________________________________________

End of Block: mechanisms

Start of Block: booster_bs

booster_bs_timing Timing

First Click (1)

Last Click (2)

Page Submit (3)

Click Count (4)

Display this question:

If booster_between = less_effective

less_eff Next, we’d like you to imagine a new hypothetical scenario:

A new and highly contagious variant of COVID-19 emerges several months from now. A new booster has been developed to combat this new variant. This new booster is safe and recommended for everyone regardless of whether they previously had any COVID-19 vaccinations.

This new booster is less effective at preventing COVID-19 infection than previous vaccines.

The government would like people to get this new booster. In this scenario, which of the following policies would you support?

Display this question:

If booster_between = as_effective

as_eff Next, we’d like you to imagine a new hypothetical scenario:

A new and highly contagious variant of COVID-19 emerges several months from now. A new booster has been developed to combat this new variant. This new booster is safe and recommended for everyone regardless of whether they previously had any COVID-19 vaccinations.

This new booster is as effective at preventing COVID-19 infection as previous vaccines.

The government would like people to get this new booster. In this scenario, which of the following policies would you support?

Display this question:

If booster_between = more_effective

more_eff Next, we’d like you to imagine a new hypothetical scenario:

A new and highly contagious variant of COVID-19 emerges several months from now. A new booster has been developed to combat this new variant. This new booster is safe and recommended for everyone regardless of whether they previously had any COVID-19 vaccinations.

This new booster is more effective at preventing COVID-19 infection than previous vaccines.

The government would like people to get this new booster. In this scenario, which of the following policies would you support?

|  |
| --- |

booster_1 The government makes this new booster freely available to all eligible adults.

- I would support this (1)
- I would oppose this (2)

|  |
| --- |

booster_2 The government promotes this new booster using advertisements.

- I would support this (1)
- I would oppose this (2)

|  |
| --- |

booster_3 The government sends text messages to eligible adults reminding them that this new booster is available.

- I would support this (1)
- I would oppose this (2)

|  |
| --- |

booster_4 The government gives a tax break to eligible adults who get this new booster.

- I would support this (1)
- I would oppose this (2)

|  |
| --- |

booster_5 The government allows employers to require their eligible employees to get this new booster.

- I would support this (1)
- I would oppose this (2)

|  |
| --- |

booster_6 The government requires eligible adults to show proof that they got this new booster before they can enter certain indoor places such as restaurants, gyms, and theatres.

- I would support this (1)
- I would oppose this (2)

|  |
| --- |

booster_7 The government fines eligible adults who refuse this new booster.

- I would support this (1)
- I would oppose this (2)

End of Block: booster_bs

Start of Block: manip_check

|  |  |
| --- | --- |

booster_manip In the hypothetical scenario you just read, how effective was this new booster at preventing COVID-19 infection compared to previous vaccines?

- Less effective than previous vaccines (1)
- As effective as previous vaccines (2)
- More effective than previous vaccines (3)

End of Block: manip_check

Start of Block: post-treatment

post_t Timing

First Click (1)

Last Click (2)

Page Submit (3)

Click Count (4)

post There is a great deal of uncertainty about how the future might look with the coronavirus still in circulation. Please describe what you expect to happen with the coronavirus pandemic in 2022 in the text box below.

________________________________________________________________

________________________________________________________________

________________________________________________________________

________________________________________________________________

________________________________________________________________

| Page Break |  |
| --- | --- |

future_scenario_1_t Timing

First Click (1)

Last Click (2)

Page Submit (3)

Click Count (4)

|  |
| --- |

future_scenario_1 Next, we'd like to know what you think will happen in the future with the coronavirus (COVID-19). On a scale of 0-10, where 0 means ‘completely unlikely to happen’ and 10 means ‘completely likely to happen’, please tell us how likely you think the following will happen **in the United Kingdom**. 

There are no ‘right’ or ‘wrong’ answers. Please answer as honestly as you can.

|  | Completely unlikely to happen | Completely likely to happen |
| --- | --- | --- |

|  | 0 | 1 | 2 | 3 | 4 | 5 | 6 | 7 | 8 | 9 | 10 |
| --- | --- | --- | --- | --- | --- | --- | --- | --- | --- | --- | --- |

| Compared to today, the number of COVID-19 related fatalities per day in the UK will be higher in 12 months. () |  |
| --- | --- |
| Compared to today, the number of COVID-19 cases per day in the UK will be higher in 12 months. () |  |
| COVID-19 vaccines available in the UK in 12 months will be more effective than the vaccines available today. () |  |
| People in the UK will be legally required to show proof of vaccination to access indoor public places in 12 months. () |  |
| Compared to today, public compliance with COVID-19 safety measures recommended by the UK government will be higher in 12 months. () |  |
| In 12 months, the UK government will recommend at least one new booster shot. () |  |

| Page Break |  |
| --- | --- |

future_scenario_2_t Timing

First Click (1)

Last Click (2)

Page Submit (3)

Click Count (4)

|  |
| --- |

future_scenario_2 Again, we'd like to know what you think will happen in the future with the coronavirus (COVID-19). On a scale of 0-10, where 0 means ‘completely unlikely to happen’ and 10 means ‘completely likely to happen’ please tell us how likely you think the following will happen **in the United Kingdom**. 

There are no ‘right’ or ‘wrong’ answers. Please answer as honestly as you can.

|  | Completely unlikely to happen | Completely likely to happen |
| --- | --- | --- |

|  | 0 | 1 | 2 | 3 | 4 | 5 | 6 | 7 | 8 | 9 | 10 |
| --- | --- | --- | --- | --- | --- | --- | --- | --- | --- | --- | --- |

| In 12 months, COVID-19 booster shots will be mandatory in the UK. () |  |
| --- | --- |
| There will be more restrictions on international travel to and from the UK in 12 months. () |  |
| Compared to today, people in the UK will socialise more in-person in 12 months. () |  |
| People in the UK will be legally required to wear masks in indoor public places in 12 months. () |  |
| Compared to today, people in the UK will work more often from home in 12 months. () |  |

End of Block: post-treatment

Start of Block: serious

|  |
| --- |

serious_now How serious of a threat do you think COVID-19 is right now?

- Very serious (1)
- Somewhat serious (2)
- Not very serious (3)
- Not at all serious (4)

| Page Break |  |
| --- | --- |

|  |  |
| --- | --- |

serious_past Compared to when COVID-19 first emerged, how serious of a threat do you think COVID-19 is right now?

- COVID-19 is more serious today than when it first emerged. (1)
- COVID-19 is as serious today as when it first emerged. (2)
- COVID-19 is less serious today than when it first emerged. (3)

| Page Break |  |
| --- | --- |

|  |  |
| --- | --- |

serious_future Compared to today, how serious of a threat do you think COVID-19 will be one year from now?

- COVID-19 will be more serious one year from now than it is today. (1)
- COVID-19 will be as serious one year from now as it is today. (2)
- COVID-19 will be less serious one year from now than it is today. (3)

End of Block: serious

Start of Block: feedback

feedback Do you have any comments on the survey? Please let us know about any problems you had or aspects of the survey that were confusing.

________________________________________________________________

________________________________________________________________

________________________________________________________________

________________________________________________________________

________________________________________________________________

End of Block: feedback
