# Supplementary material for: What nudges you to take a vaccine? Understanding behavioural drivers of COVID-19 vaccinations using large-scale experiments in the G-7 countries
Source: Health Psychol Behav Med. 2025 Apr 16;13(1):2490550. doi: 10.1080/21642850.2025.2490550 (PMC12004716; doi:10.1080/21642850.2025.2490550)
Supplement: Data Paper Appendix 2.docx [file RHPB_A_2490550_SM5915.docx]

**APPENDIX 2 – Codebook
List of variables (as per survey)
version 1.0**

| Variable name | Survey measurement item | Measurement type (original as per survey) | Coded as | Source |
| --- | --- | --- | --- | --- |
| ETHICS- | | | | |
| ethics | Thank you for taking the time to participate in this study.    Please note that you need to be 18+ and speak English fluently, otherwise please don't partake. The study should take around 15 minutes to complete. In the study, you will be asked a series of questions about vaccines. You will be paid for your participation in the survey. Just make sure to read all the instructions carefully and try your best.    NOTE: You can withdraw from the study at any stage without providing an explanation. Your privacy is very important, so we always use anonymised data. Results from this work may be written up for publication in a peer reviewed journal. However, individual data will never be published, and we will not hold personal identifiers. This project is in line with the ethical guidelines established by the Research Ethics Committee of King’s College London.    For more details about this research project, please see this information sheet.    If you have any questions you would like to ask before starting the survey, please feel free to contact Professor Peter John, King’s College London: [redacted for privacy concerns].    Please note in this survey we would like to ask some questions that may be perceived as sensitive, such as gender, ethnicity, political orientation, and religion. Providing information in response to these questions is entirely voluntary and you may withdraw your consent at any time. If you are happy to participate, please choose "I give my consent to participate in this research study." | Binary: I do not give my consent to participate in this research study, I give my consent to participate in this research study | Binary  0 - "I DO NOT give my consent to participate in this research study."  1 - "I give my consent to participate in this research study." | Standard KCL ethics form. |
| yob | What is your age? Please enter as a number (e.g., 25). | Continuous | Values represent the respondents age in years | Lazarus et al., (2020) |
| _age | CONSTRUCTED VARIABLE - yob variable sorted in to six distinct categories | Categorical: 18-24, 25-34, 35-44, 45-54, 55-64, 65+ | 1 - "18-24"  2 - "25-34"  3 - "35-44"  4 - "45-54"  5 - "55-64"  6 - "65+" | Used by the authors in publications associated with this dataset. |
| SCREENER QUESTION 1 | | | | |
| screen1 | Help us keep track of who is paying attention - please select “somewhat disagree” in the options below. | Multiple choice question: Strongly disagree, somewhat disagree, neither agree not disagree, somewhat agree, strongly agree | 1 - "Strongly disagree"  2 - "Somewhat disagree"  3 - "Neither agree nor disagree"  4 - "Somewhat agree"  5 - "Strongly agree" | Source: Arechar et al., (2019) |
| country | Do you currently live in [country]? | Binary; yes, no | 0 - “No”  1 - “Yes” | Constructed by authors for purpose of this study. |
| DEMOGRAPHICS | | | | |
| _respondent_country | CONSTRUCTED VARIABLE - The respondent’s country derived from which version of the survey was used. | Categorical (Canada, France, Germany, Italy, Japan, UK, USA) | String |  |
| _G7 | CONSTRUCTED VARIABLE - The respondent’s country derived from which version of the survey was used. | Categorical (Canada, France, Germany, Italy, Japan, UK, USA) | 1 - “Canada”  2 - “France”  3 - “Germany”  4 - “Italy”  5 - “Japan”  6 - “UK”  7 - ”USA” |  |
| subnat_region | Which region do you currently live in? | Categorical (varies per country). Canada: Alberta, British Columbia, Manitoba, New Brunswick, Newfoundland and Labrador, Nova Scotia, Ontario, Prince Edward Island, Quebec, Saskatchewan, Nunavut, Yukon; UK: Cymru Wales, East, Midlands, East of England, London, North East & Cumbria, North West, Northern Ireland, Scotland, South East, South West, West Midlands, Yorkshire & the Humber; France: Alsace, Aquitaine, Auvergne, Basse-Normandie, Bourgogne, Bretagne, Centre, Champagne-Ardenne, Corse, Franche-Comte, Haute-Normandie, Languedoc-Roussillon, Limousin, Lorraine, Midi-Pyrences, Nord-Pas-de-Calais, PaysdelaLoire, Picardie, Poitou-Charentes, Provence-Alpes-Cyted’Azur, Rhyne-Alpes, ´ele-de-France; Germany: Bayern, Berlin, Brandenburg, Bremen, Hamburg, Hessen, Mecklenburg-Vorpommern, Niedersachsen, Nordrhein-Westfalen, Rheinland-Pfalz, Saarland, Sachsen, Sachsen-Anhalt, Schleswig-Holstein, Thoringen; Italy: Abruzzo, Basilicata, Calabria, Campania, Emilia-Romagna, Friuli-Venezia Giulia, Lazio, Liguria, Lombardia, Marche, Molise, Piemonte, Puglia, Sardegna, Sicilia, Toscana, Trentino-Alto Adige, Umbria, Valle d’Aosta, Veneto; Japan: Aichi, Akita, Aomori, Chiba, Ehime, Fukui, Fukuoka, Fukushima, Gifu, Gunma, Hiroshima, Hokkaido, Hyogo, Ibaraki, Ishikawa, Iwate, Kagawa, Kagoshima, Kanagawa, Kochi, Kumamoto, Kyoto, Mie, Miyagi, Miyazaki, Nagano, Nagasaki, Nara, Niigata, Oita, Okayama, Okinawa, Osaka, Saga, Saitama, Shiga, Shimane, Shizuoka, Tochigi, Tokushima, Tokyo, Tottori, Toyama, Wakayama, Yamagata, Yamaguchi, Yamanashi US: Alabama, Alaska, Arizona, Arkansas, California, Colorado, Connecticut, Delaware, Florida, Georgia, Hawaii, Idaho, Illinois, Indiana, Iowa, Kansas, Kentucky, Louisiana, Maine, Maryland, Massachusetts, Michigan, Minnesota, Mississippi, Missouri, Montana, Nebraska, Nevada, New Hampshire, New Jersey, New Mexico, New York, North Carolina, North Dakota, Ohio, Oklahoma, Oregon, Pennsylvania, Rhode Island, South Carolina, South Dakota, Tennessee, Texas, Utah, Vermont, Virginia, Washington, West Virginia, Wisconsin, Wyoming | String | Modified based on similar item from Bell et al., (2020); Malik et al., (2020); Sherman et al., (2020); Caserotti et al., (2021); British Social Attitudes (BSA)  Clery et al., (2021); Lazarus et al., (2020) |
| gender | Are you… | Categorical: A man, a woman, non-binary, another gender (please specify, with text entry), prefer not to answer | String | Modified based on similar item from Wong et al., (2021); Lazarus et al., (2020); de Figueiredo & Larson (2021); Solís Arce et al., (2021); Detoc et al, (2020); Gagneux-Brunon et al., (2021); Caserotti et al., (2021); Malik et al., (2020); Sherman et al., (2020) |
| education | What is the highest level of education you have achieved? | Categorical (varies per country): Canada: No schooling, Some elementary school, Completed elementary school, Some secondary/ high school, Completed secondary/ high school, Some technical, community college, CEGEP, College Classique, Completed technical, community college, CEGEP, College Classique, Some university, Bachelor's degree, Master's degree, Professional degree or doctorate, Prefer not to answer; | String | Modified based on Wong et al., (2021); Lazarus et al., (2020); de Figueiredo & Larson (2021); Solís Arce et al., (2021); Malik et al., (2020); Sherman et al., (2020); World Value Survey  Haerpfer et al., (2020). |
|  |  | France: École primaire, Collège, Lycée d`enseignement général et technologique, Lycée professionnel, Études supérieures courtes (Bac +2), Études supérieures longues (Bac +3/4/5 – Grandes écoles/Universités), Doctorat / Post-doctorat, Préfère ne pas répondre; |  |  |
|  |  | Germany: Grundschule, Hauptschule, Realschule, Gymnasium/ Berufliches Gymnasium/ Fachgymnasium, Fachoberschule, Fachschule, Berufsschule, Berufsfachschule, Technische Hochschule/ Pädagogische Hochschule, Kunsthochschule/ Musikhochschule, Fachhochschule, Universität, Technische Universität, Ich möchte keine Angabe machen; |  |  |
|  |  | Italy: Scuola elementare, Scuola media inferiore, Istituto professionale, Scuola superiore, Università, Master, Dottorato, Preferisco non rispondere; |  |  |
|  |  | Japan: Elementary school, Lower secondary school, Upper secondary school, Specialized training college, Junior college, University, Prefer not to answer; |  |  |
|  |  | UK: Combined Junior and Infant School, Infant School, Junior School, Comprehensive School, Comprehensive School (GCSE), Secondary Modern (GCSE), Grammar School (GCSE), City Technology College (GCSE), Sixth Form, College, Institution of Higher education, Open College, College of Technology, Institute, Teacher Training College, University, Open University, Prefer not to answer; |  |  |
|  |  | US: Completed some high school, High school graduate, Completed some college, College degree, Completed some postgraduate, Master's degree, Doctorate, law or professional degree, Prefer not to answer |  |  |
| _education_binary | CONSTRUCTED VARIABLE - Education harmonised across countries into a binary indicator of higher education yes/no | Binary: No higher education/no higher education | 0 - No higher education  1 - Higher education |  |
| citizen | Are you a citizen of [the United Kingdom]? | Binary: yes/no. | -99 - Missing  0 - “No”  1 - “Yes” | World Value Survey  Haerpfer et al., (2020) |
| parent_screen | Are you a parent or guardian to any children? | Binary: yes/no | 0 - “No”  1 - “Yes” | Bell et al., (2020) |
| children | How many children are you the parent or guardian of? | Dropdown list (continuous): 0 to 20 or more | -99 - Missing  0 to 20 or more | Bell et al., (2020). Modified version of World Value Survey  Haerpfer et al., (2020) |
| children_u18 | Are there any children under the age of 18 living in your household? | Dropdown list (continuous): 0 to 20 or more | -99 - Missing  0 to 20 or more | Imperial College London survey (2021) |
| ethnicity | Which of the following best describes your ethnicity? | Categorical (varies per country): Canada: Arab, Asian, Black, Indigenous (e.g. First Nations, Métis, Inuit, etc.), Latino/Latina, South Asian (e.g., East Indian, Pakistani, Sri Lankan, etc.), Southeast Asian (e.g., Vietnamese, Cambodian, Laotian, Thai, etc.), West Asian (e.g., Iranian, Afghan, etc.), White, Other (please specify); | Respondents were able to indicate multiple ethnicities. These are represented by a series of variables (ethnicity_1-ethnicity_17) indicating whether a particular ethnicity was chosen. Due to country level differences, we present these variables in string format. Users of these data are advised to check the meaning of each variable BY COUNTRY before deciding on the best way to encode these data for their own purposes. | Bell et al., (2020). Modified version of Sherman et al., (2020); Malik et al., (2020); World Value Survey  Haerpfer et al., (2020); Lazarus et al., (2020) |
|  |  | France/ Germany/ Italy: omitted due to sensitivity concerns |  |  |
|  |  | UK: White: British, White: Irish, White: Other, Mixed: White and Black Caribbean, Mixed: White and Black African, Mixed: White and Asian, Mixed: Other mixed background, Black or Black British: African, Black or Black British: Caribbean, Black or Black British: Any other Black background, Asian or Asian British: Indian, Asian or Asian British: Pakistani, Asian or Asian British: Bangladeshi, Asian or Asian British: Other Asian background, Chinese, Other ethnic group not represented by these options (please specify); |  |  |
|  |  | US: White, Hispanic or Latino, Black or African-American, American Indian or Alaska Native, Asian, Native Hawaiian / Pacific Islander, Other (specify) |  |  |
| employment_1 | What is your employment status? Please select as many as applicable. | Binaryl: Working for pay full-time | 0 - “No”  1 - “Yes” | Modified version of Imperial College London survey (2021); de Figueiredo & Larson, (2021); Malik et al., (2020); Sherman et al., (2020); World Value Survey  Haerpfer et al., (2020); |
| employment_2 | What is your employment status? Please select as many as applicable. | Binary: Working for pay part-time | 0 - “No”  1 - “Yes” | Modified version of Imperial College London survey (2021); de Figueiredo & Larson, (2021); Malik et al., (2020); Sherman et al., (2020); World Value Survey  Haerpfer et al., (2020); |
| employment_3 | What is your employment status? Please select as many as applicable. | Binary: Self-employed | 0 - “No”  1 - “Yes” | Modified version of Imperial College London survey (2021); de Figueiredo & Larson, (2021); Malik et al., (2020); Sherman et al., (2020); World Value Survey  Haerpfer et al., (2020); |
| employment_4 | What is your employment status? Please select as many as applicable. | Binary: Retired | 0 - “No”  1 - “Yes” | Modified version of Imperial College London survey (2021); de Figueiredo & Larson, (2021); Malik et al., (2020); Sherman et al., (2020); World Value Survey  Haerpfer et al., (2020); |
| employment_5 | What is your employment status? Please select as many as applicable. | Binary: Unemployed/Looking for work | 0 - “No”  1 - “Yes” | Modified version of Imperial College London survey (2021); de Figueiredo & Larson, (2021); Malik et al., (2020); Sherman et al., (2020); World Value Survey  Haerpfer et al., (2020); |
| employment_6 | What is your employment status? Please select as many as applicable. | Binary: Student | 0 - “No”  1 - “Yes” | Modified version of Imperial College London survey (2021); de Figueiredo & Larson, (2021); Malik et al., (2020); Sherman et al., (2020); World Value Survey  Haerpfer et al., (2020); |
| employment_7 | What is your employment status? Please select as many as applicable. | Binary: Caring for family | 0 - “No”  1 - “Yes” | Modified version of Imperial College London survey (2021); de Figueiredo & Larson, (2021); Malik et al., (2020); Sherman et al., (2020); World Value Survey  Haerpfer et al., (2020); |
| employment_8 | What is your employment status? Please select as many as applicable. | Binary: Other (Please specify) | 0 - “No”  1 - “Yes” | Modified version of Imperial College London survey (2021); de Figueiredo & Larson, (2021); Malik et al., (2020); Sherman et al., (2020); World Value Survey  Haerpfer et al., (2020); |
| income | What was your total household income, before taxes, for the year 2021? | Categorical (varies per country): Canada: No income, $1 - $7,500, $7,501 - $15,000, $15,001 - $30,000, $30,001 - $45,000, $45,000 - $60,000, $60,001 - $90,000, $90,001 - $110,000, $110,001 - $150,000, $150,001 - $200,000, More than $200,000, Prefer not to answer; France/ Germany/ Italy: No income, 1€ - 5,250€, 5,251€ - 10,500€, 10,501€ - 21,000€, 21,001€ - 31,500€, 31,501€ - 42,000€, 42,001€ - 63,000€, 63,001€ - 77,000€, 77,001€ - 105,000€, 105,001 - 140,000€, More than 140,000€, Don't know / prefer not to answer; Japan: No income, ¥1 - ¥675,000, ¥675,001 - ¥1,350,000, ¥1,350,001 - ¥2,700,000, ¥2,700,001 - ¥4,050,000, ¥4,050,001 - ¥5,400,000, ¥5,400,001 - ¥8,100,000, ¥8,100,001 - ¥9,950,000, ¥9,950,001 - ¥13,550,000, ¥13,550,001 - ¥18,700,000, More than ¥18,700,000, Prefer not to answer; UK: No income, £1 - £4,400, £4,401 - £8,800, £8,801 - £17,600, £17,601 - £26,400, £26,401 - £35,200, £35,201 - £52,800, £52,801 - £64,500, £64,501 - £88,000, £88,001 - £117,300, More than £117,300, Don't know / prefer not to answer; US: No income, $1 - $6,000, $6,001 - $12,000, $12,001 - $24,000, $24,001 - $36,000, $36,001 - $48,000, $48,001 - $72,000, $72,001 - $80,000, $80,001 - $120,000, $120,001 - $160,000, More than $160,000, Don't know / prefer not to answer | String | Bell et al., (2020). Modified version of Lazarus et al., (2020). |
| urban_rural | Which of the following best describes the place where you now live… | Categorical: a large city, a suburb near a large city, a small city, a town, a rural area | 1 - "A rural area"  2 - "A town"  3 - "A small city"  4 - "A suburb near large city  5 - "A large city" | Miller et al., (2012) |
| religiosity | In your life, you would say religion is: | Categorical (1-4): Very important, somewhat important, not very important, not at all important | 1- "Not at all important"  2 - "Not very important"  3 - "Somewhat important"  4 - "Very important" | Modified version of item from Sherman et al., (2020); World Value Survey  Haerpfer et al., (2020); |
| POLITICAL PREFERENCES | | | | |
| partyid | In politics, do you usually think of yourself as a(n): | Categorical (varies per country): Canada: Liberal, Conservative, NDP, Bloc Québécois, Green, People's Party of Canada, Another party (specify), No party, Don't know; | String | British Social Attitudes (BSA)  Clery et al., (2021) |
|  |  | France: La République En Marche! (LREM), Les Républicains (LR), Parti socialiste (PS), Mouvement démocrate (MoDem), La France Insoumise (FI), Parti communiste français (PCF), Rassemblement national (RN), Parti républicain, radical et radical-socialiste (RAD), Les Centristes (LC), Force européenne démocrate (FED), La Gauche moderne (LGM), Mouvement des progressistes (MDP), Debout la France (DLF), Mouvement républicain et citoyen (MRC), Cap21 (LRC-Cap21), Europe Écologie Les Verts, Comités Jeanne, Another party (specify); |  |  |
|  |  | Germany: Social Democratic Party of Germany, Christian Democratic Union of Germany, Christian Social Union in Bavaria, Alliance 90/The Greens, Free Democratic Party, Alternative for Germany, The Left, South Schleswig Voters' Association, German Centre-Party, Die Partei, Free Voters, Ecological Democratic Party, Volt Germany, Pirate Party Germany, Family Party of Germany, Liberal Conservative Reformers, Another party (specify); |  |  |
|  |  | Italy: Five Star Movement, Lega per Salvini Premier, Democratic Party, Froza Italia, Brothers of Italy, Another party (specify), No party, Don't know; Japan: Liberal Democratic Party, Constitutional Democratic Party, Komeito; |  |  |
|  |  | Japan Innovation Party, Democratic Party for the People, Japan Communist Party, Reiwa Shinsengumi, Social Democratic Party, Party of Hope, The Party to Protect the People from NHK, Another party (specify); |  |  |
|  |  | UK: Conservative Party, Labour Party, Scottish National Party, Liberal Democrats, Democratic Unionist Party, Sinn Féin, Plaid Cymru, Social Democratic and Labour Party, Green Party, Alliance Party of Northern Ireland, Another party (specify); |  |  |
|  |  | US: Republican, Democrat, Independent, Another party (specify), No party, Don't know |  |  |
| partyid_strength | How strongly [selected partyid] do you feel? | Categorical (1-4): Very strongly, fairly strongly, not very strongly, don’t know | -99 - "Don't know"  1 - "Not very strongly" 2 2 - "Fairly strongly"  3 - "Very strongly" | British Social Attitudes (BSA)  Clery et al., (2021) |
| BELIEFS AND ATTITUDES | | | | |
| risk | On a scale of 0-10, where 0 means you are ‘completely unwilling to take risks’ and 10 means you are ‘very willing to take risks’ in general, how willing or unwilling are you to take risks? | Categorical (0-10 Likert scale): 0= completely unwilling to take risks, 10= very willing to take risks. | 0 - Completely unwilling to take risks  10 - Very willing to take risks. | Dohmen et al. (2011) in the German Socio-Economic Panel (SOEP) |
| lr_scale | In political matters, people talk of the 'left' and the 'right'. How would you place your views on this scale, generally speaking? | Categorical (0-10 Likert scale): 0= left, 10= right. | 0 - Left  10 - Right | European Social Survey (ESS); Eurobarometer; World Values Survey (WVS); German Socio-Economic Panel (SOEP) |
| pol_system | On a scale of 0-10, where 0 means you have ‘no confidence at all’ and a 10 means you have a ‘great deal of confidence’, how much confidence do you have in the following institutions? | Categorical (0-10 Likert scale); 0= no confidence at all, 10= great deal of confidence. | 0 - No confidence at all,  10 - Great deal of confidence. | Adapted from “trust in institutions” as relevant for this study from European Social Survey (ESS), World Values Survey (WVS), and Eurobarometer. |
| pol_system_1 | The government |  |  |  |
| pol_system_2 | Political parties |  |  |  |
| pol_system_3 | Parliament |  |  |  |
| pol_system_4 | The armed forces |  |  |  |
| pol_system_5 | The press |  |  |  |
| pol_system_6 | Television |  |  |  |
| pol_system_7 | Labour unions |  |  |  |
| pol_system_8 | The police |  |  |  |
| pol_system_9 | The courts |  |  |  |
| pol_system_10 | The civil service |  |  |  |
| pol_system_11 | Universities |  |  |  |
| populism | How much do you agree or disagree with the following statements? | Categorical (1-5 Likert scale); 1 = "Strongly disagree", 2 = "Somewhat disagree", 3 = "Neither agree nor disagree", 4 = "Somewhat agree", 5 = "Strongly agree" | 1 - "Strongly disagree"  2 - "Somewhat disagree"  3 - "Neither agree nor disagree"  4 - "Somewhat agree"  5 - "Strongly agree" | Largely based on populist scales developed first in Akkerman,Mudde, and Zaslove (2014). |
| populism_1 | The politicians in the House of Commons need to follow the will of the people |  |  |  |
| populism_2 | The people, and not politicians, should make our most important policy decisions |  |  |  |
| populism_3 | The political differences between the elite and the people are larger than the differences among the people |  |  |  |
| populism_4 | I would rather be represented by a citizen than by a specialized politician |  |  |  |
| populism_5 | Elected officials talk too much and take too little action |  |  |  |
| populism_6 | What people call “compromise” in politics is really just selling out on one’s principles |  |  |  |
| COVID-19 HISTORY | | | | |
| had_c19 | Have you been infected with coronavirus (COVID-19) since the start of the pandemic? | Categorical: yes/no/unsure | 0 - "No"  1 - "Unsure"  2 - "Yes" | Modified version of item from Malik et al., (2020) |
| fam_c19 | Besides you, has anyone in your household been infected with coronavirus (COVID-19) since the start of the pandemic? | Categorical: yes/no/unsure | 0 - "No"  1 - "Unsure"  2 - "Yes" | Modified version of item from Lazarus et al., (2020) |
| vaccinated_c19 | Have you received a coronavirus (COVID-19) vaccine? Please do not include any information about booster shots received. We will ask about booster shots later. | Categorical: Yes, I have received a one-shot vaccine, Yes, I have received the first dose of a two-shot vaccine, Yes, I have received two doses of a two-shot vaccine, No, I have not received any vaccine doses. | String | COVID-19 Trends and Impact Survey (CTIS) (Wave 8, V2) |
| _vaccine | CONSTRUCTED VARIABLE - Did the respondent receive a full/partial/no dose? | Categorical: No dose/Partial dose/Complete dose | 0 - “No dose”  1 - “Partial dose”  2 - “Complete dose” | Constructed from vaccinated_c19 |
| no_vaccine_c19 | You selected ‘No, I have not received any vaccine doses’ in the previous step. Please select all reasons that apply to your answer. | Multiple responses allowed. Answers provided in a series of binary variables. 0- the associated reason does not apply, 1 - the associated reason applies. | 0 - Reason does not apply  1 - Reason applies | COVID-19 Trends and Impact Survey (CTIS) (Wave 8, V5a) |
| no_vaccine_c19_1 | A coronavirus vaccine was not available to me |  |  |  |
| no_vaccine_c19_2 | I was not eligible for a coronavirus vaccine |  |  |  |
| no_vaccine_c19_3 | I did not want to receive a coronavirus vaccine |  |  |  |
| no_vaccine_c19_4 | I did not have time to access a coronavirus vaccine. |  |  |  |
| booster_c19 | Have you received a coronavirus (COVID-19) booster vaccine? | Categorical: Yes, I have received one or more shots of a coronavirus booster vaccine, No, I have not received a coronavirus booster vaccine. | -99 - "Missing"  0 - "No, I have not received a coronavirus (COVID-19) booster vaccine."  1 - "Yes, I have received one or more shots of a coronavirus (COVID-19) booster vaccine." | Adapted based on a question on vaccine uptake from CTIS. |
| booster_second | How many booster shots have you received? | Categorical: I have not received a booster shot, 1 booster shot, 2 booster shots, 3 booster shots, 4 booster shots, more than 4 booster shots. | 0 - "I have not received a booster shot."  1 - "1 booster shot."  2 - "2 booster shots"  3 - "3 booster shots"  4 - "4 booster shots"  5 - "More than 4 booster shots." | Adapted based on a question on vaccine uptake from CTIS. |
| no_booster_c19 | You selected ‘No, I have not received a coronavirus (COVID-19) booster vaccine’ in the previous step. Please select all reasons that apply to your answer. | Multiple responses allowed. Answers provided in a series of binary variables. 0- the associated reason does not apply, 1 - the associated reason applies. | 0 - Reason does not apply  1 - Reason applies | Adapted based on a question on vaccine uptake from CTIS. |
| no_booster_c19_1 | A coronavirus (COVID-19) booster was not available to me. |  |  |  |
| no_booster_c19_2 | I was not eligible for a coronavirus (COVID-19) booster. |  |  |  |
| no_booster_c19_3 | I did not want to take a coronavirus (COVID-19) booster. |  |  |  |
| no_booster_c19_4 | I did not have time to access a coronavirus (COVID-19) booster. |  |  |  |
| actions_om_c19 | On a scale of 0-10, where 0 means 'never' and 10 means 'always', how frequently do you currently engage in the following actions or behaviours in response to the coronavirus (COVID-19)? | Categorical (0-10 Likert scale); 0= never, 10= always, including not applicable. | 0 - Never  10 - Always, including not applicable. | Constructed using insights from CTIS as well as Imperial College London’s COVID-19 Behavioural Tracker. |
| actions_om_c19_1 | Wear a face mask in indoor public spaces |  |  |  |
| actions_om_c19_2 | Wear a face mask in outdoor public spaces |  |  |  |
| actions_om_c19_3 | Work, or attend school, from home |  |  |  |
| actions_om_c19_4 | Avoid going to pubs/bars/restaurants |  |  |  |
| actions_om_c19_5 | Social distance in indoor public areas |  |  |  |
| actions_om_c19_6 | Avoid small indoor gatherings |  |  |  |
| actions_om_c19_7 | Avoid large indoor gatherings |  |  |  |
| actions_om_c19_8 | Social distance in outdoor public areas |  |  |  |
| actions_om_c19_9 | Avoid small outdoor gatherings |  |  |  |
| actions_om_c19_10 | Avoid large outdoor gatherings |  |  |  |
| trust_vaccines | On a scale of 0-10, where 0 means ‘strongly disagree’ and 10 means ‘strongly agree’, how much do you agree or disagree, with the following statements? | Categorical (0-10 Likert scale); 0= strongly disagree, 10= strongly agree. | 0 - Strongly disagree  10 - Strongly agree. | Modified version of item from Sherman et al., (2020) and the Imperial College London COVID-19 Behavioural Tracker. |
| trust_vaccines_1 | Vaccination is generally good for building immunity against the novel coronavirus that causes COVID |  |  |  |
| trust_vaccines_2 | A vaccine will protect those who receive it from possible health effects of coronavirus |  |  |  |
| trust_vaccines_3 | Without a vaccine, I am likely to catch coronavirus |  |  |  |
| trust_vaccines_4 | I am worried about potential side effects of a coronavirus vaccine |  |  |  |
| trust_vaccines_5 | Receiving a coronavirus vaccine has improved my sense of belonging to my community |  |  |  |
| trust_vaccines_6 | Too much fuss is being made about the risk of coronavirus. |  |  |  |
| prevent_infection | To the best of your knowledge, how effective are currently available vaccines at preventing COVID-19 infection? | Categorical: Very effective, Somewhat effective, Not very effective, Not at all effective | 1 - "Not at all effective"  2 - "Not very effective"  3 - "Somewhat effective"  4 - "Very effective" | WAVE 2 ONLY |
| ignore_community | On a scale of 0-10, where 0 means ‘strongly disagree’ and 10 means ‘strongly agree’, how much do you agree or disagree with the following statement? Politicians usually ignore my community. | Categorical (0-10 Likert scale); 0= strongly disagree, 10= strongly agree. | 0 - Strongly disagree  10 - Strongly agree. | Adapted from World Value Survey  Haerpfer et al., (2020) |
| lives_over_liberty | On a scale of 0-10, where 0 means ‘strongly disagree’ and 10 means ‘strongly agree’, how much do you agree or disagree with the following statement?  Saving lives is more important than personal liberty. | Categorical (0-10 Likert scale); 0= strongly disagree, 10= strongly agree. | 0 - Strongly disagree  10 - Strongly agree. | Constructed item based on similar questions around trade-offs between public safety and individual freedoms. |
| trust_vaccines2 | Generally speaking, would you say that coronavirus (COVID-19) vaccines can be trusted? | Binary: Yes, coronavirus (COVID-19) vaccines can be trusted/ No, coronavirus (COVID-19) vaccines cannot be trusted. | 0 - "No, coronavirus (COVID-19) vaccines cannot be trusted."  1 - "Yes, coronavirus (COVID-19) vaccines can be trusted." | Modified version of item in the Imperial College London COVID-19 Behavioural Tracker |
| trust_who | Which of the following sources would you trust MOST to help you decide whether you would get a COVID-19 vaccine and/or the booster? | Multiple Choice Question: Your doctor or healthcare provider, Your co-worker, Your employer, [head of government], Your local public health authority, Your friends or family, Your neighbours, Your local community, Your pastor, priest, or other religious leader, Other. | String | Adapted version of the trust in institutions question for the COVID-19 vaccine and/or booster. |
| _trust_who | CONSTRUCTED VARIABLE - Values of the original trust_who variable standardised across countries so that there is a single category for “Head of government” | Multiple Choice Question: Your doctor or healthcare provider, Your co-worker, Your employer, Head of government, Your local public health authority, Your friends or family, Your neighbours, Your local community, Your pastor, priest, or other religious leader, Other. | 1 - "Your co-worker"  2 - "Your doctor or healthcare provider"  3 - "Your employer"  4 - "Your friends or family"  5 - "Your local community"  6 - "Your local public health authority"  7 - "Your neighbours"  8 - "Your pastor, priest, or other religious leader"  9 - "Other (please specify)" |  |
| MEDIA CONSUMPTION | | | | |
| covid_news_source | What is your primary source for news on the coronavirus (COVID-19) pandemic? | Categorical: Newspapers, Television news, Radio, Social media, WhatsApp, WeChat. | -99 - Missing  1 - "Newspapers (websites or in print)"  2 - "Radio"  3 - "Social media"  4 - "Television news"  5 - "WeChat"  6 - "WhatsApp" | Adapted from the COVID-19 Trends and Impact Survey (CTIS). |
| covid_news_volume | How often have you read, listened to, or watched news related to the coronavirus (COVID-19) pandemic over the past week? | Categorical: Several times a day, Daily, almost every day, A few times, Once, Never. | -99 - Missing  1 - "Never"  2 - "Once"  3 - "A few times"  4 - "Almost every day"  5 - "Daily"  6 - "Several times a day" | COVID-19 Trends and Impact Survey (CTIS) |
| SCREENER QUESTION 2 | | | | |
| screen_2 | People are very busy these days and many do not have time to follow what goes on in the government. We are testing whether people read questions. To show that you’ve read this much, answer both “Extremely interested” and “Very interested”: | Multiple responses allowed. Answers provided in a series of binary variables. 0- the respondent did not provide the associated answer, 1 - the respondent provided the associated answer. | 0- The respondent did not provide the associated answer  1 - The respondent provided the associated answer. | Source: Aronow, et al., (2020) |
| screen_2_1 | "Extremely disinterested" |  |  |  |
| screen_2_2 | "Very disinterested" |  |  |  |
| screen_2_3 | "Somewhat disinterested" |  |  |  |
| screen_2_4 | "Neither disinterested nor interested" |  |  |  |
| screen_2_5 | "Somewhat interested" |  |  |  |
| screen_2_6 | "Very interested" |  |  |  |
| screen_2_7 | "Extremely interested" |  |  |  |
| CONJOINT EXPERIMENT*  (Large conjoint studies were conducted to understand vaccination preferences for COVID-19 prior to our study. Major studies that inspired our work include: Genie et al., (2020); Borriello et al., (2021); H. Chu & S. Liu (2021); B. Craig (2021); Dong et al., (2020); Duch et al., (2021); Eshun-Wilson et al., (2021); Huang et al., (2021); K. Kawata & M. Nakabayashi (2021); Kreps et al., (2021); S. Kreps & D. Kriner (2021); Leng et al., (2021); J. Luyten, S. Tubeuf & R. Kessels (2020); R. McPhedran & B. Toombs (2021); M. Motta (2021); Schwarzinger et al., (2021); P. Vanhuysse, M. Jankowski & M. Tepe (2021); Wang et al., (2021); Yuen et al., (2021))  *The conjoint experiment (attributes and levels) were modified in wave 2 of the study. Please refer to the transparency changes document on OSF for more details on specific modifications made and its rationale. | | | | |
| prefer | ROUND 1 - Which is your preferred choice of future vaccine policy? | Multiple choice question: Vaccine Policy A, Vaccine Policy B | -99 - Missing  1 - Vaccine A  2 - Vaccine B | Standard Conjoint Question |
| support1 | ROUND 1 - On a scale of 0-10, where 0 means you ‘definitely do not support’ this vaccine policy, and 10 means you ‘definitely support' this vaccine policy, how would you rate each vaccine policy? | Continuous (0-10 Likert scale); 0= definitely do not support, 10= definitely support. | 0 - Definitely do not support  10 - Definitely support. |  |
| support1_1 | Vaccine A |  |  |  |
| support1_2 | Vaccine B |  |  |  |
| prefer2 | ROUND 2 - Which is your preferred choice of future vaccine policy? | Multiple choice question: Vaccine Policy A, Vaccine Policy B | -99 - Missing  1 - Vaccine A  2 - Vaccine B |  |
| support2 | ROUND 2 - On a scale of 0-10, where 0 means you ‘definitely do not support’ this vaccine policy, and 10 means you ‘definitely support' this vaccine policy, how would you rate each vaccine policy? | Continuous (0-10 Likert scale); 0= definitely do not support, 10= definitely support. | 0 - Definitely do not support  10 - Definitely support. |  |
| support2_1 | Vaccine A |  |  |  |
| support2_2 | Vaccine B |  |  |  |
| prefer3 | ROUND 3 - Which is your preferred choice of future vaccine policy? | Multiple choice question: Vaccine Policy A, Vaccine Policy B | -99 - Missing  1 - Vaccine A  2 - Vaccine B |  |
| support3 | ROUND 3 - On a scale of 0-10, where 0 means you ‘definitely do not support’ this vaccine policy, and 10 means you ‘definitely support' this vaccine policy, how would you rate each vaccine policy? | Continuous (0-10 Likert scale); 0= definitely do not support, 10= definitely support. | 0 - Definitely do not support  10 - Definitely support. |  |
| support3_1 | Vaccine A |  |  |  |
| support3_2 | Vaccine B |  |  |  |
| STRINGENCY between subjects experiment*  *The stringency experiment (attributes and levels) were modified in wave 2 of the study. Please refer to the transparency changes document on OSF for more details on specific modifications made and its rationale. | | | | |
| tradeoff | Imagine the following scenario: In October of 2022, a new variant emerges which, like Omicron, is highly contagious. New vaccine boosters are developed. These boosters provide ${e://Field/effective} protection against infection from the new variant.  The government in your country would like individuals to take this booster shot. Which of the following policies would you support? | Multiple choice question: yes/no/unsure | String | Constructed by authors for the specific purpose of this study. |
| tradeoff_1 | No one should be forced to take the booster, but it should be available to anyone who wants it |  |  |  |
| tradeoff_2 | Those who do not take the booster should be stopped from entering any indoor public spaces and/or using public transport |  |  |  |
| tradeoff_3 | Employers should require their employees to get the booster |  |  |  |
| tradeoff_4 | Those who do not take the booster should be fined by the government |  |  |  |
| _policystringency1 | CONSTRUCTED VARIABLE - tradeoff_1 “yes” and “unsure” collapsed (reverse coded) | Binary Variable | 0 - “Yes/Unsure”  1 - “No” |  |
| _policystringency2 | CONSTRUCTED VARIABLE - tradeoff_2 “no” and “unsure” collapsed (reverse coded) | Binary Variable | 0 - “No/Unsure”  1 - “Yes” |  |
| _policystringency3 | CONSTRUCTED VARIABLE - tradeoff_3 “no” and “unsure” collapsed (reverse coded) | Binary Variable | 0 - “No/Unsure”  1 - “Yes” |  |
| _policystringency4 | CONSTRUCTED VARIABLE - tradeoff_4 “no” and “unsure” collapsed (reverse coded) | Binary Variable | 0 - “No/Unsure”  1 - “Yes” |  |
| _policystringency | CONSTRUCTED VARIABLE - summative index of policy stringency | Summative Index (_policystringcy1 + _policystringcy2 + _policystringcy3 + _policystringcy4) | Summative Index (0-4) |  |
| effective | ASSIGNED VARIABLE | 10 percentage point increments starting at 50% and ending at 90%. | 50%, 60%, 70%, 80%, 90% |  |
| SCREENER QUESTION 3 | | | | |
| screen3 | Most modern theories of decision making recognize that decisions do not take place in a vacuum. Individual preferences and knowledge, along with situational variables can greatly impact the decision process. To demonstrate that you’ve read this much, just go ahead and select both red and green among the alternatives below, no matter what your favourite colour is. Yes, ignore the question below and select both of those options.    What is your favourite colour? | Multiple responses allowed. Answers provided in a series of binary variables. 0- the respondent did not provide the associated answer, 1 - the respondent provided the associated answer. | 0 - Respondent provide the associated answer  1 - Respondent provided associated answer | Source: Berinsky et al., (2014) |
| screen3_1 | White |  |  |  |
| screen3_2 | Black |  |  |  |
| screen3_3 | Red |  |  |  |
| screen3_4 | Pink |  |  |  |
| screen3_5 | Green |  |  |  |
| screen3_6 | Blue |  |  |  |
| NUDGE+ between subjects experiment*  *The nudge+ experiment (attributes and levels) were modified in wave 2 of the study. Please refer to the transparency changes document on OSF for more details on specific modifications made and its rationale. | | | | |
| treatment | CONSTRUCTED VARIABLE – indicates treatment status | Categorical variable - control, nudge, nudge+, think | Study 1:  1 - Control  2 - Nudge  3 - Nudge+  4 - Think  Study 2:  1 - Control  2 - Default schedule  3 - Norm Nudge  4 - Text Reminder | Constructed by authors for the specific purpose of this study. |
| annual | In this scenario, how likely is it that you would get this booster? | Categorical (1-6 Likert scale): Very Likely, somewhat likely, slightly likely, slightly unlikely, somewhat unlikely, very unlikely. | 1 - Very unlikely  2 - Somewhat unlikely  3 - Slightly unlikely  4 - Slightly likely  5 - Somewhat likely  6 - Very likely |  |
| child_all | In this scenario, if you had a child under the age of 18, how likely is it that you would allow your child to get this booster? | Categorical (1-6 Likert scale): Very Likely, somewhat likely, slightly likely, slightly unlikely, somewhat unlikely, very unlikely. | 1 - Very unlikely  2 - Somewhat unlikely  3 - Slightly unlikely  4 - Slightly likely  5 - Somewhat likely  6 - Very likely |  |
| approve | Do you approve or disapprove of the government’s action in this scenario? Please answer using the scale below, where 0 means ‘I disapprove of the government’s action’ and 10 means ‘I approve of the government’s action’. | Categorical (0-10 Likert scale): 0= I disapprove of the government’s action, 10= I approve of the government’s action. | 0 - I disapprove of the government’s action  10 - I approve of the government’s action |  |
| action | In this scenario, do you think the government is doing too little, just the right amount, or too much to manage the coronavirus (COVID-19) pandemic in your country? | Categorical: too little, just the right amount, too much | 1 - Too little  2 - Just the right amount  3 - Too much |  |
| manicheck | In this scenario, what did the government do to manage rising COVID-19 cases in your area? | Multiple choice question: The government leaves it to every adult living in your country to choose whether they should get this vaccine booster shot or not, The government announces that every adult living in your country will be automatically enrolled to receive this vaccine booster shot at a local clinic, The government announces that every living adult in your country will be required to receive this vaccine booster to travel, The government announces that it will fine adults living in your country who do not receive this vaccine booster. | String |  |
| text | Please think about the government's actions in this scenario. Do you think this approach is appropriate? Do you think this approach will work for you? In at least one or two sentences, please write down your thoughts. | Open ended text. | REDACTED for privacy concerns as responses can identify participants. | |
| moregovernment | What else would you like the government to do to manage the coronavirus (COVID-19) pandemic in the scenario described above?    In at least one or two sentences, please write down your thoughts in the text box below. | Open ended text. | REDACTED for privacy concerns as responses can identify participants. | |
| post | There is a great deal of uncertainty about how the future might look with the coronavirus still in circulation. Please describe what you expect to happen with the coronavirus pandemic in 2022 in the text box below. | Open ended text. | REDACTED for privacy concerns as responses can identify participants. | |
| future_scenario | Next, we'd like to know what you think will happen in the future with the coronavirus (COVID-19). On a scale of 0-10, where 0 means ‘completely unlikely to happen’ and 10 means ‘completely likely to happen’, please tell us how likely you think the following will happen in the United Kingdom.    There are no ‘right’ or ‘wrong’ answers. Please answer as honestly as you can. | Categorical (0-10 Likert scale): 0= I Completely unlikely to happen, 10= Completely likely to happen. | 0 - I Completely unlikely to happen  10 - Completely likely to happen. | Constructed by authors for the specific purpose of this study. |
| future_scenario_1 | In 12 months, COVID-19 booster shots will be mandatory in [country]. |  |  |  |
| future_scenario_2 | There will be more restrictions on international travel to and from [country] in 12 months. |  |  |  |
| future_scenario_3 | Compared to today, people in [country] will socialise more in-person in 12 months. People in [country] will be legally required to wear masks in indoor public places in 12 months. |  |  |  |
| future_scenario_4 | Compared to today, people in [country] will work more often from home in 12 months. |  |  |  |
| future_scenario_5 | - Compared to today, the number of COVID-19 related fatalities per day in [country] will be higher in 12 months. |  |  |  |
| future_scenario_6 | Compared to today, the number of COVID-19 cases per day in [country] will be higher in 12 months. |  |  |  |
| future_scenario_7 | COVID-19 vaccines available in [country] in 12 months will be more effective than the vaccines available today. |  |  |  |
| future_scenario_8 | People in [country] will be legally required to show proof of vaccination to access indoor public places in 12 months. |  |  |  |
| future_scenario_9 | Compared to today, public compliance with COVID-19 safety measures recommended by the [country] government will be higher in 12 months. |  |  |  |
| future_scenario_10 | In 12 months, the [country] government will recommend at least one new booster shot. |  |  |  |
| MECHANISMS EXPERIMENT  Wave 2 Only | | | | |
| _mechanism | ASSIGNED VARIABLE - Series of policy instruments for encouraging booster vaccination | Categorical Variable - Health clinics call, Health clinics text, Employers allowed to require vaccine mandates, Eligible adults fined if they do not take a booster, Government launches advertising campaign | String |  |
| mechanisms1 | How effective do you think this policy would be at encouraging people to get this new booster? | Categorical Variable: 4 point scale | 1 - "Not at all effective"  2 - "Somewhat effective"  3 - "Not very effective"  4 - "Very effective" |  |
| mechanisms2 | Which of the following statements comes closest to your view? | Categorical Variable: too little to protect people’s freedom to decide for themselves, strikes an appropriate balance between freedom to decide and encouraging people to decide, too little to encourage people to get the booster. | 1 - "This policy does too little to protect people's freedom to decide for themselves whether to get this new booster."  2 - "This policy strikes an appropriate balance between protecting people's freedom to decide for themselves whether to get this new booster and encouraging people to get this new booster."  3 - "This policy does too little to encourage people to get this new booster." |  |
| mechanism3 | Do you have any further thoughts about this policy? If yes, please feel free to share your thoughts in the space provided below. | Open-ended text | REDACTED for privacy concerns as responses can identify participants. | |
| Booster Between Subjects Experiment  Wave 2 Only | | | | |
| booster_between | ASSIGNED VARIABLE - Hypothetical vaccine effectiveness relative to real-world contemptuous  vaccines | Categorical variable - Less/As/More Effective | 1 - "Less Effective"  2 - "As Effective"  3 - "More Effective" |  |
| booster_1 | The government makes this new booster freely available to all eligible adults. | Binary Variable - I would oppose/support this | 0 - “I would oppose this”  1 - “I would support this” |  |
| booster_2 | The government promotes this new booster using advertisements. |  |  |  |
| booster_3 | The government sends text messages to eligible adults reminding them that this new booster is available. |  |  |  |
| booster_4 | The government allows employers to require their eligible employees to get this new booster. |  |  |  |
| booster_5 | The government requires eligible adults to show proof that they got this new booster before they can enter certain indoor places such as restaurants, gyms, and theatres. |  |  |  |
| booster_6 | The government fines eligible adults who refuse this new booster. |  |  |  |
| booster_manip | In the hypothetical scenario you just read, how effective was this new booster at preventing COVID-19 infection compared to previous vaccines? | Categorical variable - Less/As/More Effective | String |  |

References:

Arechar, A. A., Mosleh, M., Pennycook, G., & Rand, D. (2019). Sharing intentions in survey experiments predict actual sharing behavior on social media. *Open Science Framework*. Available here: <https://osf.io/w5987>

Aronow, P. M., Kalla, J., Orr, L., & Ternovski, J. (2020). Evidence of rising rates of inattentiveness on Lucid in 2020. *SocArXiv, 49*, 59-63. Available here: <https://osf.io/8sbe4>

Bell, S., Clarke, R., Mounier-Jack, S., Walker, J. L., & Paterson, P. (2020). Parents’ and guardians’ views on the acceptability of a future COVID-19 vaccine: A multi-methods study in England. *Vaccine, 38*(49), 7789–7798.<https://doi.org/10.1016/j.vaccine.2020.10.027>

Berinsky, A. J., Margolis, M. F., & Sances, M. W. (2014). Separating the shirkers from the workers? Making sure respondents pay attention on self‐administered surveys. *American journal of political science, 58*(3), 739-753. <https://doi.org/10.1111/ajps.12081>

Borriello, A., Master, D., Pellegrini, A., & Rose, J. M. (2021). Preferences for a COVID-19 vaccine in Australia. *Vaccine, 39*(3), 473–479.<https://doi.org/10.1016/j.vaccine.2020.12.032>

Caserotti, M., Girardi, P., Rubaltelli, E., Tasso, A., Lotto, L., & Gavaruzzi, T. (2021). Associations of COVID-19 risk perception with vaccine hesitancy over time for Italian residents. *Social Science & Medicine, 272*, 113688.<https://doi.org/10.1016/j.socscimed.2021.113688>

Chu, H., & Liu, S. (2021). Light at the end of the tunnel: Influence of vaccine availability and vaccination intention on people’s consideration of the COVID-19 vaccine. *Social Science & Medicine, 286*, 114315.<https://doi.org/10.1016/j.socscimed.2021.114315>

Clery, E., Curtice, J., Frankenburg, S., Morgan, H., & Reid, S. (Eds.). (2021). *British social attitudes: The 38th report*. National Centre for Social Research.

Craig, B. M. (2021). United States COVID-19 Vaccination Preferences (CVP): 2020 Hindsight. *The Patient - Patient-Centered Outcomes Research, 14*(3), 309–318.<https://doi.org/10.1007/s40271-021-00508-0>

Detoc, M., Bruel, S., Frappe, P., Tardy, B., Botelho-Nevers, E., & Gagneux-Brunon, A. (2020). Intention to participate in a COVID-19 vaccine clinical trial and to get vaccinated against COVID-19 in France during the pandemic. *Vaccine, 38*(45), 7002–7006.<https://doi.org/10.1016/j.vaccine.2020.09.041>

de Figueiredo, A., & Larson, H. J. (2021). Exploratory study of the global intent to accept COVID-19 vaccinations. *Communications Medicine, 1*(1), 30.<https://doi.org/10.1038/s43856-021-00027-x>

Dong, D., Xu, R., Wong, E., Hung, C., Feng, D., Feng, Z., . . . Wong, S. (2020). Public preference for COVID‐19 vaccines in China: A discrete choice experiment. *Health Expectations: An International Journal of Public Participation in Health Care and Health Policy, 23*(6), 1543–1578.<https://doi.org/10.1111/hex.13140>

Duch, R., Roope, L. S. J., Violato, M., Becerra, M. F., Robinson, T. S., Bonnefon, J.-F., Friedman, J., Loewen, P. J., Mamidi, P., Melegaro, A., Blanco, M., Vargas, J., Seither, J., Candio, P., Cruz, A. G., Hua, X., Barnett, A., & Clarke, P. M. (2021). Citizens from 13 countries share similar preferences for COVID-19 vaccine allocation priorities. *Proceedings of the National Academy of Sciences, 118*(38).<https://doi.org/10.1073/pnas.2026382118>

Eshun-Wilson, I., Mody, A., Tram, K. H., Bradley, C., Sheve, A., Fox, B., Thompson, V., & Geng, E. H. (2021). Preferences for COVID-19 vaccine distribution strategies in the US: A discrete choice survey. *PLOS ONE, 16*(8), e0256394.<https://doi.org/10.1371/journal.pone.0256394>

Global attitudes: Covid-19 vaccines | Ipsos. (2021). Retrieved November 19, 2021, from<https://www.ipsos.com/es-do/global-attitudes-covid-19-vaccine-january-2021>

Gagneux-Brunon, A., Detoc, M., Bruel, S., Tardy, B., Rozaire, O., Frappe, P., & Botelho-Nevers, E. (2021). Intention to get vaccinations against COVID-19 in French healthcare workers during the first pandemic wave: A cross-sectional survey. *Journal of Hospital Infection, 108*, 168–173.<https://doi.org/10.1016/j.jhin.2020.11.020>

Haerpfer, C., Inglehart, R., Moreno, A., Welzel, C., Kizilova, K., Diez-Medrano, J., Lagos, M., Norris, E., Ponarin, E., & Puranen, B. (Eds.). (2020). *World values survey: Round seven – Country pooled datafile*. JD Systems Institute & WVSA Secretariat.<https://doi.org/10.14281/18241.13>

Huang, W., Shao, X., Wagner, A. L., Chen, Y., Guan, B., Boulton, M. L., Li, B., Hu, L., & Lu, Y. (2021). COVID-19 vaccine coverage, concerns, and preferences among Chinese ICU clinicians: A nationwide online survey. *Expert Review of Vaccines, 20*(10), 1361–1367.<https://doi.org/10.1080/14760584.2021.1971523>

Imperial College London COVID-19 Behavioural Tracker. (2021). Covid-19: Global attitudes towards a COVID-19 vaccine. Retrieved November 19, 2021, from<https://www.imperial.ac.uk/media/imperial-college/institute-of-global-health-innovation/EMBARGOED-0502.-Feb-21-GlobalVaccineInsights_ICL-YouGov-Covid-19-Behaviour-Tracker_20210301.pdf>

Kawata, K., & Nakabayashi, M. (2021). Determinants of COVID-19 vaccine preference: A survey study in Japan. *SSM - Population Health, 15*, 100902.<https://doi.org/10.1016/j.ssmph.2021.100902>

Kreps, S., Dasgupta, N., Brownstein, J., Hswen, Y., & Kriner, D. (2021). Public attitudes toward COVID-19 vaccination: The role of vaccine attributes, incentives, and misinformation. *Npj Vaccines, 6*(1), 73.<https://doi.org/10.1038/s41541-021-00335-2>

Kreps, S., & Kriner, D. (2021). Factors influencing Covid-19 vaccine acceptance across subgroups in the United States: Evidence from a conjoint experiment. *Vaccine, 39*(24), 3250–3258.<https://doi.org/10.1016/j.vaccine.2021.04.044>

Leng, A., Maitland, E., Wang, S., Nicholas, S., Liu, R., & Wang, J. (2021). Individual preferences for COVID-19 vaccination in China. *Vaccine, 39*(2), 247–254.<https://doi.org/10.1016/j.vaccine.2020.12.009>

Lazarus, J. V., Ratzan, S. C., Palayew, A., Gostin, L. O., Larson, H. J., Rabin, K., Kimball, S., & El-Mohandes, A. (2021). A global survey of potential acceptance of a COVID-19 vaccine. *Nature Medicine, 27*(2), 225–228.<https://doi.org/10.1038/s41591-020-1124-9>

Lazarus, J. V., Ratzan, S., Palayew, A., Billari, F. C., Binagwaho, A., Kimball, S., Larson, H. J., Melegaro, A., Rabin, K., White, T. M., & El-Mohandes, A. (2020). COVID-SCORE: A global survey to assess public perceptions of government responses to COVID-19 (COVID-SCORE-10). *PLOS ONE, 15*(10), e0240011.<https://doi.org/10.1371/journal.pone.0240011>

McPhedran, R., & Toombs, B. (2021). Efficacy or delivery? An online Discrete Choice Experiment to explore preferences for COVID-19 vaccines in the UK. *Economics Letters, 200*, 109747.<https://doi.org/10.1016/j.econlet.2021.109747>

Malik, A. A., McFadden, S. M., Elharake, J., & Omer, S. B. (2020). Determinants of COVID-19 vaccine acceptance in the US. *EClinicalMedicine, 26*, 100495.<https://doi.org/10.1016/j.eclinm.2020.100495>

Motta, M. (2021). Can a COVID-19 vaccine live up to Americans’ expectations? A conjoint analysis of how vaccine characteristics influence vaccination intentions. *Social Science & Medicine (1982), 272*, 113642.<https://doi.org/10.1016/j.socscimed.2020.113642>

Pew Research Center. (2012, September 26). The state of online dating. Pew Research Center. Retrieved from<https://www.pewresearch.org/internet/2012/09/26/main-report-13/>

Schwarzinger, M., Watson, V., Arwidson, P., Alla, F., & Luchini, S. (2021). COVID-19 vaccine hesitancy in a representative working-age population in France: A survey experiment based on vaccine characteristics. *The Lancet. Public Health, 6*(4), E210–E221.<https://doi.org/10.1016/S2468-2667(21)00012-8>

Sherman, S. M., Smith, L. E., Sim, J., Amlôt, R., Cutts, M., Dasch, H., Rubin, G. J., & Sevdalis, N. (2020). COVID-19 vaccination intention in the UK: Results from the COVID-19 vaccination acceptability study (CoVAccS), a nationally representative cross-sectional survey. *Human Vaccines & Immunotherapeutics*.<https://doi.org/10.1080/21645515.2020.1846397>

Solís Arce, J. S., Warren, S. S., Meriggi, N. F., Scacco, A., McMurry, N., Voors, M., Syunyaev, G., Malik, A. A., Aboutajdine, S., Adeojo, O., Anigo, D., Armand, A., Asad, S., Atyera, M., Augsburg, B., Awasthi, M., Ayesiga, G. E., Bancalari, A., Björkman Nyqvist, M., … Omer, S. B. (2021). COVID-19 vaccine acceptance and hesitancy in low- and middle-income countries. *Nature Medicine, 27*(8), 1385–1394.<https://doi.org/10.1038/s41591-021-01454-y>

Vanhuysse, P., Jankowski, M., & Tepe, M. (2021). Vaccine alliance building blocks: A conjoint experiment on popular support for international COVID-19 cooperation formats. *Policy Sciences, 54*(3), 493–506.<https://doi.org/10.1007/s11077-021-09435-1>

Wang, J., Wagner, A. L., Chen, Y., Jaime, E., Hu, X., Wu, S., Lu, Y., Ruan, Y., & Pan, S. W. (2021).

Public willingness to accept COVID-19 vaccination in China: A nationwide survey. *Human Vaccines & Immunotherapeutics, 17*(1), 221–227.<https://doi.org/10.1080/21645515.2020.1848611>

World Health Organization. (2021). *WHO global strategy for vaccination and immunization*. Retrieved from<https://www.who.int/strategies/vaccines>
